# Supplementary material for: Mesothelioma and Radical Surgery 2 (MARS 2): protocol for a multicentre randomised trial comparing (extended) pleurectomy decortication versus no (extended) pleurectomy decortication for patients with malignant pleural mesothelioma
Source: BMJ Open. 2020 Sep 1;10(9):e038892. doi: 10.1136/bmjopen-2020-038892 (PMC7467531; doi:10.1136/bmjopen-2020-038892)
Supplement: Author's manuscript [file bmjopen-2020-038892.draft_revisions.pdf]

BMJ Open is committed to open peer review. As part of this commitment we make the peer review history of every article we publish publicly available.

When an article is published we post the peer reviewers' comments and the authors' responses online. We also post the versions of the paper that were used during peer review. These are the versions that the peer review comments apply to.

The versions of the paper that follow are the versions that were submitted during the peer review process. They are not the versions of record or the final published versions. They should not be cited or distributed as the published version of this manuscript.

BMJ Open is an open access journal and the full, final, typeset and author-corrected version of record of the manuscript is available on our site with no access controls, subscription charges or pay-per-view fees (http://bmjopen.bmj.com).

If you have any questions on BMJ Open's open peer review process please email info.bmjopen@bmj.com

BMJ Open

Mesothelioma and Radical Surgery 2 (MARS 2): protocol for a multicentre randomised trial comparing (extended) pleurectomy decortication versus no (extended) pleurectomy decortication for patients with malignant pleural mesothelioma

Journal:	*BMJ Open*
Manuscript ID	bmjopen-2020-038892
Article Type:	Protocol
Date Submitted by the Author:	27-Mar-2020

Complete List of Authors:	Lim, Eric; Royal Brompton and Harefield NHS Trust, Academic Division of Thoracic Surgery<br>Darlison, Liz; University Hospitals of Leicester NHS Trust, The Glenfield Hospital; Mesothelioma UK, c/o Glenfield Hospital<br>Edwards , John ; Sheffield Teaching Hospitals NHS Foundation Trust, Cardiothoracic Centre<br>Elliott, Daisy; University of Bristol, Population Health Sciences<br>Fennell, DA; University Hospitals of Leicester NHS Trust,<br>Popat, Sanjay; Royal Marsden NHS Foundation Trust, Department of Medicine<br>Rintoul, Robert; Papworth Hospital NHS Foundation Trust, Department of Thoracic Oncology<br>Waller , David ; Barts Health NHS Trust, Department of Thoracic Surgery<br>Ali, Clinton ; Gartnavel General Hospital, The Beatson West of Scotland Cancer Centre<br>Bille, Andrea; Guy's and Saint Thomas' Hospitals NHS Trust, Thoracic Surgery<br>Fuller , Liz; South Tyneside NHS Foundation Trust, Respiratory Medicine<br>Ionescu, Andreea; Aneurin Bevan Health Board, Lung Cancer<br>Keni, Manjusha; Derby Teaching Hospitals NHS Foundation Trust, Oncology<br>Kirk , Alan ; Golden Jubilee National Hospital, Department of Thoracic Surgery<br>Koh, Pek; New Cross Hospital, Department of Oncology<br>Lau, Kelvin ; Barts Health NHS Trust, Department of Thoracic Surgery<br>Mansy, Talal; South Tees Hospitals NHS Foundation Trust, Oncology<br>Maskell, Nick; University of Bristol, Academic Respiratory Unit, School of Clinical Sciences; North Bristol NHS Trust, Respiratory Research Unit<br>Milton , Richard ; Leeds Teaching Hospitals NHS Trust, Thoracic Surgery Department<br>Muthukumar, Dakshinamoorthy; Colchester Hospital University NHS Foundation Trust, Oncology<br>Pope , Tony ; Clatterbridge Cancer Centre NHS Foundation Trust, Clatterbridge Cancer Centre<br>Roy , Amy ; University Hospitals Plymouth NHS Trust, Plymouth Oncology Centre<br>Shah, Riyaz; Maidstone and Tunbridge Wells NHS Trust

Shamash , Jonathan; Barts Health NHS Trust, Department of Medical Oncology
Tasigiannopoulos, Zacharias ; Norfolk and Norwich University Hospital NHS Trust, The Oncology Care Team
Taylor, Paul; The Christie NHS Foundation Trust, Medical Oncology
Treece , Sarah; North West Anglia NHS Foundation Trust, Heamatology and Oncology Unit
Ashton, Kate; University of Bristol, Bristol Trials Centre (CTEU)
Harris, Rosie; University of Bristol, Bristol Trials Centre (CTEU)
Joyce, Katherine; University of Bristol, Bristol Trials Centre (CTEU)
Warnes, Barbara; University of Bristol, Bristol Trial Centre (CTEU)
Mills, Nicola; University of Bristol, Population Health Sciences
Stokes, Elizabeth; University of Oxford, Health Economics Research Centre Nuffield Department of Population Health
Rogers, Chris; University of Bristol, Bristol Trials Centre (CTEU)

Keywords: ONCOLOGY, CHEMOTHERAPY, Thoracic surgery < SURGERY, THORACIC MEDICINE

SCHOLARONE™ Manuscripts

I, the Submitting Author has the right to grant and does grant on behalf of all authors of the Work (as defined in the below author licence), an exclusive licence and/or a non-exclusive licence for contributions from authors who are: i) UK Crown employees; ii) where BMJ has agreed a CC-BY licence shall apply, and/or iii) in accordance with the terms applicable for US Federal Government officers or employees acting as part of their official duties; on a worldwide, perpetual, irrevocable, royalty-free basis to BMJ Publishing Group Ltd ("BMJ") its licensees and where the relevant Journal is co-owned by BMJ to the co-owners of the Journal, to publish the Work in this journal and any other BMJ products and to exploit all rights, as set out in our licence.

The Submitting Author accepts and understands that any supply made under these terms is made by BMJ to the Submitting Author unless you are acting as an employee on behalf of your employer or a postgraduate student of an affiliated institution which is paying any applicable article publishing charge ("APC") for Open Access articles. Where the Submitting Author wishes to make the Work available on an Open Access basis (and intends to pay the relevant APC), the terms of reuse of such Open Access shall be governed by a Creative Commons licence – details of these licences and which Creative Commons licence will apply to this Work are set out in our licence referred to above.

Other than as permitted in any relevant BMJ Author's Self Archiving Policies, I confirm this Work has not been accepted for publication elsewhere, is not being considered for publication elsewhere and does not duplicate material already published. I confirm all authors consent to publication of this Work and authorise the granting of this licence.

For peer review only

Mesothelioma and Radical Surgery 2 (MARS 2): protocol for a multicentre randomised trial comparing (extended) pleurectomy decortication versus no (extended) pleurectomy decortication for patients with malignant pleural mesothelioma

Eric Lim[1]	e.lim@rbht.nhs.uk
Liz Darlison[2]	liz.darlison@uhl-tr.nhs.uk
John G Edwards[3]	john.edwards3@nhs.net
Daisy Elliott[4]	daisy.elliott@bristol.ac.uk
Dean A Fennell[5]	df132@leicester.ac.uk
Sanjay Popat[6]	sanjay.popat@rmh.nhs.uk
Robert Rintoul[7]	robert.rintoul@nhs.net
David Waller[8]	david.waller1@nhs.net
Clinton Ali[9]	clinton.ali@ggc.scot.nhs.uk
Andrea Bille[10]	andrea.bille@gstt.nhs.uk
Liz Fuller[11]	liz.fuller@stft.nhs.uk
Andreea A Ionescu[12]	alina.ionescu2@wales.nhs.uk
Manjusha Keni[13]	manjusha.keni@nhs.net
Alan Kirk[14]	Alan.Kirk@gjnh.scot.nhs.uk
Pek Koh[15]	pek.koh@nhs.net
Kelvin Lau[8]	kelvin.lau@bartshealth.nhs.uk
Talal Mansy[16]	talal.mansy@stees.nhs.uk
Nick A Maskell[17]	nick.maskell@bristol.ac.uk
Richard Milton[18]	richard.milton@leedsth.nhs.uk
Dakshinamoorthy Muthukumar[19]	muthu.kumar@colchesterhospital.nhs.uk
Tony Pope[20]	tony.pope@nhs.net
Amy Roy[21]	amyroy@nhs.net
Riyaz Shah[22]	riyaz.shah@nhs.net
Jonathan Shamash[23]	jonathan.shamash@bartshealth.nhs.uk
Zacharias Tasigiannopoulos[24]	zacharias.tasigiannopoulos@nnuh.nhs.uk
Paul Taylor[25]	paul.taylor@uhsm.nhs.uk
Sarah Treece[26]	sarah.treece@pbh-tr.nhs.uk
Kate Ashton[27]	kate.ashton@bristol.ac.uk
Rosie A Harris[27]	rosie.harris@bristol.ac.uk
Katherine Joyce[27]	katie.joyce@bristol.ac.uk
Barbara Warnes[27]	barbara.warnes@bristol.ac.uk

For peer review only

Nicola Mills[4] nicola.mills@bristol.ac.uk

Elizabeth Stokes[28] elizabeth.stokes@dph.ox.ac.uk

Chris A Rogers on behalf of the MARS 2 Trialists*[27] chris.rogers@bristol.ac.uk

CORRESPONDING AUTHOR:

Professor Eric Lim

Academic Division of Thoracic Surgery

The Royal Brompton Hospital

Sydney Street

London SW3 6NP

United Kingdom

Tel: +44 (0)207 351 8591

Fax: +44 (0)207 351 8560

Email: e.lim@rbht.nhs.uk

1. Academic Division of Thoracic Surgery, The Royal Brompton and Harefield NHS Foundation Trust, London, UK
2. Institute for Lung Health, University Hospitals of Leicester, Leicester, UK
3. Cardiothoracic Centre, Sheffield Teaching Hospitals NHS Foundation Trust, Sheffield, UK
4. Population Health Sciences, University of Bristol, Bristol, UK
5. Cancer Research Centre, University Hospitals of Leicester NHS Trust, Leicester Royal Infirmary, Leicester, UK
6. Department of Medicine – Lung, Institute of Cancer Research, The Royal Marsden NHS Foundation Trust, Fulham Road, London, UK
7. Department of Oncology, University of Cambridge and Papworth Trials Unit Collaboration, Royal Papworth Hospital NHS Foundation Trust, Cambridge, UK
8. Department of Thoracic Surgery, Barts Health NHS Trust, The Royal London Hospital, London, UK
9. The Beatson West of Scotland Cancer Centre, Gartnavel General Hospital, Glasgow, UK
10. Thoracic Surgery, Guy's and St Thomas's NHS Foundation Trust, Guy's Hospital, London, UK
11. Respiratory Medicine, South Tyneside General District Hospital, South Shields, UK
12. Lung Cancer, Aneurin Bevan Health Board (Royal Gwent Hospital), Newport, UK
13. Oncology, Derby Teaching Hospitals NHS Foundation Trust, Derby, UK
14. Department of Thoracic Surgery, Golden Jubilee National Hospital, Glasgow, UK

For peer review only - http://bmjopen.bmj.com/site/about/guidelines.xhtml

15. Department of Oncology, Royal Wolverhampton NHS Trust, Wolverhampton, UK
16. Oncology, South Tees Hospitals NHS Foundation Trust, Middlesbrough, UK
17. Academic Respiratory Unit, University of Bristol, Bristol, UK
18. Thoracic Surgery Department, Leeds Teaching Hospitals NHS Trust, Leeds, UK
19. Oncology, Colchester Hospital University NHS Foundation Trust, Colchester, UK
20. The Clatterbridge Cancer Centre NHS Foundation Trust, Wirral, UK
21. Plymouth Oncology Centre, University Hospitals Plymouth NHS Trust, Plymouth, UK
22. Kent Oncology Centre, Maidstone and Tunbridge Wells NHS Trust, Tunbridge Wells, UK
23. Department of Medical Oncology, St Bartholomew's Hospital, London, UK
24. The Oncology Care Team Norfolk & Norwich University Hospitals NHS Foundation Trust, Norwich, UK
25. Manchester Lung Cancer Group, Manchester University NHS Foundation Trust, Manchester, UK
26. Heamatology and Oncology Unit, North West Anglia NHS Foundation Trust, Peterborough City Hospital, Peterborough, UK
27. Bristol Trials Centre Clinical Trials and Evaluation Unit, Bristol Medical School, University of Bristol, Bristol, UK
28. Health Economics Research Centre, Nuffield Department of Population Health, University of Oxford, Oxford, UK

For peer review only

WORD COUNT: 3739 (excluding title page, abstract, references, figures and tables)

Number of figures: 1

Number of tables: 1

KEYWORDS: Randomised controlled trial; mesothelioma; surgery; radical surgery; pleurectomy decortication; cancer

For peer review only - http://bmjopen.bmj.com/site/about/guidelines.xhtml

ABSTRACT

Introduction: Mesothelioma remains a lethal cancer. To date, systemic therapy with pemetrexed and a platinum drug remains the only licenced standard of care. As the median survival for mesothelioma patients is 12.1 months, surgery is an important consideration to improve survival and/or quality of life. Currently, only two surgical trials have been performed which found that neither extensive (extra-pleural pneumonectomy) or limited (partial pleurectomy) surgery improved survival (although there was some evidence of improved quality of life). Therefore, clinicians are now looking to evaluate pleurectomy decortication, the only radical treatment option left.

Methods and analysis: The MARS 2 study is a UK multicentre open parallel group randomised controlled trial comparing the effectiveness and cost-effectiveness of surgery - (extended) pleurectomy decortication - versus no surgery for the treatment of pleural mesothelioma. The study will test the hypothesis that surgery and chemotherapy is superior to chemotherapy alone with respect to overall survival. Secondary outcomes include health related quality of life, progression free survival, measures of safety (adverse events) and resource use to 2 years. The QuinteT Recruitment Intervention is integrated into the trial to optimise recruitment.

Ethics and dissemination: Research ethics approval was granted by London - Camberwell St. Giles Research Ethics Committee (reference 13/LO/1481) on 7th November 2013. We will submit the results for publication in a peer-reviewed journal.

Trial registration numbers: ISRCTN – ISRCTN44351742 and ClinicalTrials.gov – NCT02040272.

For peer review only

For peer review only - http://bmjopen.bmj.com/site/about/guidelines.xhtml

ARTICLE SUMMARY

Strengths and limitations of this study

- Pleurectomy decortication is currently offered to patients with mesothelioma on the United Kingdom National Health Service, but it is unknown whether it is a clinically beneficial or cost-effective treatment option. MARS 2 is the first randomised controlled trial to compare this type of surgery with no surgery in this patient population.
- Surgical quality assurance measures will be implemented to ensure that the intervention will be delivered at centres with expertise.
- Patients may come with a pre-conceived perception that surgery will be beneficial, which can lead to crossovers (i.e. patients allocated to no surgery may go on to seek surgery elsewhere). The integrated Quintet Recruitment Intervention supports recruitment staff in responding to patient preferences and conveying balanced information.
- It is not possible to blind participants or the study team, but the primary outcome (survival) is objective.
- Patient pathways vary at different sites. Some flexibility has been worked into the protocol to allow for this.

INTRODUCTION

In the United Kingdom (UK), approximately 2,500 patients are diagnosed each year with pleural mesothelioma,[1] a treatment resistant and lethal cancer of the membranes lining the outer surface of the lung and the inside of the chest wall primarily due to asbestos exposure. Deaths are increasing yearly and are estimated to peak this year.[2] So far, most treatments have proven ineffective. The current standard of care, consisting of 4 to 6 cycles of platinum and pemetrexed chemotherapy, as recommended by the National Institute for Health and Care Excellence (NICE)[3], has been associated with only an additional 3 months of survival.[4] As the median survival for mesothelioma patients is 12.1 months,[4] surgery to remove as much of the disease as possible remains an important consideration to improve survival and/or health related quality of life (HRQoL).[5]

Pleurectomy decortication is the most common surgical procedure for mesothelioma worldwide and is defined as parietal and visceral pleurectomy to remove all gross tumour without diaphragm or pericardial resection.[6] ‘Extended’ pleurectomy decortication can also be carried out, when parietal and visceral pleurectomy is undertaken to remove all gross

For peer review only - http://bmjopen.bmj.com/site/about/guidelines.xhtml

tumour, including the resection of the diaphragm and/or pericardium. The other main types of surgery for mesothelioma are: i) extra-pleural pneumonectomy, which is defined as en bloc resection of the parietal and visceral pleura with the ipsilateral lung, pericardium, and diaphragm (in cases where the pericardium and/or diaphragm are not involved by tumour, these structures may be left intact) and partial pleurectomy, which is the partial removal of parietal and/or visceral pleura for diagnostic or palliative purposes but leaving gross tumour behind.[6]

So far, no advantage, in terms of survival, has been observed with any type of surgery in randomised controlled trials (RCTs) conducted to date. The MARS feasibility study (ISRCTN95583524), a trial of extra-pleural pneumonectomy with adjuvant haemothorax irradiation, concluded that surgery was unlikely to offer either an improvement to survival or HRQoL and possibly harmed patients.[7] MesoVATS (ISRCTN34321019) concluded that partial pleurectomy did not improve survival, although it showed that patients in the better prognostic group, had improved HRQoL after 6 months.[8]

Suitable patients, both in the UK and internationally, are currently offered pleurectomy decortication as it is considered to carry less morbidity compared to the more extensive extra-pleural pneumonectomy but still achieves complete macroscopic resection which partial pleurectomy does not.[9-11] However, we do not know if pleurectomy decortication in conjunction with chemotherapy will improve survival compared to the current standard of care (chemotherapy alone). In the absence of RCTs, pleurectomy decortication may continue to be offered despite a lack of high-quality evidence of clinical efficacy or any evidence on cost-effectiveness.

Aims and objectives

MARS 2 is a UK wide multicentre RCT which will test the hypothesis that (extended) pleurectomy decortication and chemotherapy is superior to chemotherapy alone with respect to overall survival for patients with pleural mesothelioma.

Specific objectives are to estimate:

A. The difference between groups in overall survival.
B. The difference between groups with respect to a range of secondary outcomes including HRQoL, progression free survival and measures of safety (adverse health events).

For peer review only - http://bmjopen.bmj.com/site/about/guidelines.xhtml

C. The cost effectiveness of (extended) pleurectomy decortication compared to no surgery.

METHODS AND ANALYSIS

Trial design

MARS 2 is a multicentre, non-blinded parallel two-group, pragmatic RCT of surgery and chemotherapy versus chemotherapy alone for suitable patients with mesothelioma.

An internal pilot funded by Cancer Research UK (award ref: C27967/A15895) and coordinated by the Papworth Trials Unit Collaboration, demonstrated the feasibility of recruitment across 14 medical sites and 2 joint medical and surgical sites of excellence, as the target of 50 participants recruited within a 24 month period was achieved.

Since the end of the pilot phase in December 2016 an additional 8 medical, 1 surgical and 2 joint medical and surgical sites have been opened for the full trial. In addition, the full trial will provide recruiting sites with the support of an integrated QuinteT Recruitment Intervention (QRI)[12-14] to optimise recruitment and retention.

Setting, centre and surgeon eligibility

This study is taking place in National Health Service (NHS) secondary care centres, including teaching and district general hospitals.

To be eligible as a medical site, the centre must:

i) be an NHS Trust with access to a multidisciplinary team (MDT) to discuss patients with mesothelioma;
ii) have a track record of treating patients with mesothelioma

To be eligible as a surgical site, the centre must:

i) be an NHS Trust with an established mesothelioma MDT;
ii) have a minimum of 2 named mesothelioma surgeons participating in the trial.

All surgeons participating in the full trial must be accredited by; i) self-reporting a minimum of 5 cases in which they have performed (extended) pleurectomy decortication; ii) observing the procedure being undertaken at an established MARS 2 surgical site; iii) having a surgeon from the pilot phase observe their first MARS 2 procedure undertaken; and iv)

For peer review only - http://bmjopen.bmj.com/site/about/guidelines.xhtml

having one randomly selected MARS 2 operation between procedures 5 and 10 observed by a surgeon from the pilot phase to ensure fidelity.

Patients from all medical (only) sites are referred to a trial-accredited surgical site for computed tomography (CT) assessment of eligibility, further discussion about the study, and surgery (if randomised to this group).

Trial population

The target population are patients with a diagnosis of epithelioid, sarcomatoid or biphasic mesothelioma. Patients will be eligible to take part if ALL of the following apply:

- Adult aged ≥ 16 years of age;
- Tissue (cytology or histology) confirmed epithelioid, sarcomatoid or biphasic mesothelioma, as reviewed by MDT to be of sufficient certainty to recommend chemotherapy as treatment;
- Disease confined to one hemi-thorax based on CT assessment;
- Disease deemed surgically resectable by a surgeon at a MARS 2 surgical site;
- Deemed fit for surgery by a surgeon at a MARS 2 surgical site;
- Capacity to provide written informed consent to participate in the trial.

Patients will not be eligible if they have:

- Severe shortness of breath (Eastern Cooperative Oncology Group (ECOG) status ≥ 2; or pre-operative forced expiratory volume after one second (FEV1) or transfer factor of the lung for carbon monoxide (TLco) less than 20%);
- Severe heart failure (NYHA III or IV, or ejection fraction less than 30% by echocardiogram);
- End stage kidney failure requiring dialysis;
- Liver failure (e.g. encephalopathy and/or coagulation abnormalities);
- Any other serious concomitant disorder that would compromise participant safety during surgery;
- Prisoner;
- Patient lacks capacity to consent;
- Existing co-enrolment in another interventional study that aims to improve survival.

Patient approach, consent and randomisation

For peer review only - http://bmjopen.bmj.com/site/about/guidelines.xhtml

The local research team at the medical site will take written informed consent from participants. In addition to the main study, the team may also seek consent for audio-recording of consultations and participation in interviews, for QRI purposes. Participants will then receive two cycles of chemotherapy (standard care) and have a further CT scan to confirm eligibility (i.e. disease still resectable) before being randomised, using a secure web-based randomisation system (Sealed Envelope https://sealedenvelope.com).

Participants will be randomised in a 1:1 ratio. Minimisation (with a random component) will be applied for selected baseline variables (age, performance status and cell type) that influence survival, in addition to stratification by recruiting site to ensure that the cohorts are as balanced as possible.

Trial interventions

Patients will be randomised to receive one of the following interventions:

- *Pleurectomy decortication and chemotherapy;* two cycles of platinum and pemetrexed chemotherapy followed by surgery and then up to four cycles of the same chemotherapy.
- *Chemotherapy alone (control intervention);* Up to six cycles of platinum and pemetrexed chemotherapy alone (current standard of care).

The trial schema is illustrated in figure 1.

After randomisation, any changes in the choice of chemotherapy, addition of other agents or entry into therapeutic trials (e.g. immunotherapies) will be permitted for patients with progressive disease. At the time of trial design, there was no national consensus on post-operative prophylactic radiotherapy so it was decided that irradiation to thoracic procedure sites may be undertaken for MARS 2 patients. Patients in both groups can also receive further surgery, including thoracic, if it is without radical intent. The aim is to conduct a pragmatic trial whilst closely monitoring uptake of additional therapies, studies or surgeries in order to account for them in the trial analyses, if required.

Primary and secondary outcomes

The primary outcome is survival. All participants will be followed up to the end of the trial (minimum of two years after randomisation).

For peer review only - http://bmjopen.bmj.com/site/about/guidelines.xhtml

Secondary outcomes have been selected to assess the efficacy of the two approaches. Secondary outcomes are 1) progression free survival to the end of the trial (minimum of two years after randomisation); 2) serious adverse health events to two years after randomisation; 3) disease specific and generic HRQoL using the following validated questionnaires: European Organisation for Research and Treatment of Cancer Quality of Life Questionnaire (EORTC QLQ-C30) – to assess the HRQoL of cancer patients; and EuroQol EQ-5D-5L[15 16] – a widely used generic measure of HRQoL (both of these will be measured at baseline, pre-randomisation, and 6 weeks, 6, 12, 18 and 24 months post-randomisation) and 4) healthcare resource use to the end of the study: chemotherapy cycles and initial surgical admission (for chemotherapy plus surgery group), and further resources measured at 6 weeks post-randomisation then every 6 months, with a final follow up at the end of the study if not followed up in the previous 4 months.

Data collection

The schedule for data collection for the study is shown in table 1. Data will be collected onto purpose-designed case report forms (CRFs) and participant completed questionnaires and entered onto a bespoke database for data cleaning and analysis. Access to the database will be via a secure password-protected web-interface hosted on an NHS server. Data about adverse events will be collected and reported in accordance with Sponsor's and regulatory requirements.

For peer review only

For peer review only - http://bmjopen.bmj.com/site/about/guidelines.xhtml

Table 1 Data collection

	Pre-randomisation				Post-randomisation							
	Screening	Baseline	2 cycles chemo-therapy	End of chemo-therapy cycle 2	Surgical admission *	Up to 4 cycles chemo-therapy	Follow-up					
							6 w	6 m	12 m	18 m	24 m	Every 6 m and end of trial**
Screening log	✓			✓								
CT scan	✓***			✓								
Informed consent		✓										
Demography, medical history, blood test results		✓										
Lung function tests	✓	✓****										
HRQoL		✓		✓			✓	✓	✓	✓	✓	
Chemotherapy treatment given***			✓			✓						
Surgery and in hospital post-operative data*****					✓							
Adverse events					✓		✓	✓	✓	✓	✓	
Patient reported resource and health service use							✓	✓	✓	✓	✓	✓

* Patients allocated to surgery and/or receiving surgery only
** If not within previous 4 months
*** Previous CT scan to be used (not to be done again specifically for the trial protocol)
**** Only one assessment of lung function is needed so if this has been done prior to screening there is no need for another test at baseline
***** Including resource and health service use

For peer review only

Risk of bias

Participants and clinical personnel cannot be blinded to allocation due to the nature of the study intervention. However, standard local protocols will be followed in terms of patient care. The patient information leaflet and conversations with MARS 2 site staff will describe and balance the potential benefits and risks of both having and not having surgery. Therefore, this approach will reduce participant's expectations that one or other treatment protocol will lead to a more favourable result.

In addition, the study's primary outcome is an objective measure (survival), and clear definitions of each secondary outcome measure will be provided to trial personnel. The HRQoL follow-up questionnaires may be more at risk of bias than other measures, but patients will not have had this surgery previously and as such, should not have any expectation regarding its effect on their HRQoL. Missing outcome data will be minimised, as survival and progression free survival data can be obtained from hospital records. Losses to follow up will be minimised by maintaining regular contact with participants (by telephone and post) to complete follow-up questionnaires. Non-adherence to randomised allocation will be documented. Bias in the reported results will be minimised by having pre-specified outcomes in the trial protocol and a pre-specified analysis plan.

Sample size

The total sample size has been set at 328 participants (164 per group). The patients randomised in the pilot trial will contribute to the total sample size. The study will have 80% power to detect a hazard ratio (HR) of 0.7 at 5% statistical significance (2-sided), modelled on a published assumption of a median survival time of 16.8 months in mesothelioma patients who were fit enough to receive surgery, but did not have it[17] and allowing for 10% cross-over from the medical to surgery groups (as noted in previous trials such as MARS 1[7]). Cross-over will be minimised through instruction (i.e. recruit only patients who have equipoise from the outset) and education.

The relative difference of 30% (HR 0.7) was regarded as the minimally important difference for patients and clinicians to choose surgery given the risks of the procedure. The figure was chosen by the trial's patient and public involvement (PPI) group. The possibility that survival could be worse with surgery was also discussed, and a relative difference of 30% also regarded as an appropriate difference to indicate harm, therefore a two-tailed test for superiority was agreed.

Patient and Public Involvement

Patient and public representatives were involved from inception and advised on the trial design of MARS 2, the identification of the choice of the primary outcome and defined the minimally important difference in relative survival.

The study team have continuing engagement with the Royal Brompton Hospital Cancer Consortia PPI group that consists of patients and carers who have undergone surgery for lung cancer and mesothelioma to advise on patient orientated questions that arise from the

For peer review only

trial conduct. One patient from the PPI group, a mesothelioma survivor, has agreed to sit on the Trial Steering Committee. The PPI group will also be involved in the dissemination of study results.

Integrated QuinteT Recruitment Intervention (QRI)

Recruitment to RCTs can be challenging[18], particularly for surgical trials.[19] An integrated QRI will therefore be employed during the main study phase to optimise recruitment and retention. The aim of the QRI is to understand the recruitment process and how it operates in clinical centres, so that sources of recruitment difficulties can be identified, and suggestions made to change aspects of design, conduct, organisation or training.

A multi-faceted, flexible approach will be used to investigate site-specific or wider recruitment obstacles. These will comprise the following:

- Mapping of eligibility and recruitment pathways to collate basic data about the levels of eligibility and recruitment, and identify points at which patients opt in or out of the trial;
- In-depth, semi-structured interviews with a purposive sample of staff members involved with aspects of trial design/management and recruitment across centres, and patients eligible for recruitment to the trial. Interviews will explore participants' perspectives of the trial, views on the presentation of study information, understanding of trial processes (e.g. randomisation), and reasons underlying decisions to accept or decline the trial. In addition, interviews with staff and other individuals involved in the trial will explore perspectives on the trial design and protocol; views about the evidence on which the trial is based; perceptions of uncertainty/equipoise for themselves and their colleagues; methods for identifying eligible patients; views on eligibility, and examples of actual recruitment successes and difficulties. Interview topic guides will be used to ensure similar topic areas are covered across interviews, while still providing the scope for participants to raise issues of pertinence to them.
- Audio recording of consultations between healthcare staff and potentially eligible patients across centres to understand the recruitment process at each centre and to identify and investigate the challenges to recruitment. The QRI researcher will listen to and qualitatively analyse the appointments, documenting instances such as unclear, insufficient or imbalanced information provision and unintentional transferring of clinician treatment preferences to patients.

For peer review only - http://bmjopen.bmj.com/site/about/guidelines.xhtml

- Observation of Trial Management Group (TMG) and investigator meetings to gain an overview of trial conduct and overarching challenges (logistical issues, etc.).

An account of the anonymised findings from all the data will be fed back to the Chief Investigator and TMG. The data will be used by the QRI team to provide supportive and confidential individual and group feedback to recruiters to help them to communicate equipoise, balance treatment options and explain to patients the benefits and purposes of trial participation, whilst optimising informed consent.

Statistical analyses

The data will be analysed for randomised patients according to intention to treat and follow Consolidated Standards of Reporting Trials (CONSORT) guidelines. Analyses will be adjusted for site and for design factors included in the cohort minimisation (e.g. age, performance status and cell type).

Survival time and progression free survival time from randomisation will be compared using survival methods, allowing for censoring of any participant who is either alive or lost to follow-up at the end of the follow-up period. Patient reported outcome scores (HRQoL EQ-5D-5L and QLQ-C30) will be compared using a mixed regression model, adjusted for baseline measures where appropriate. Changes in treatment effect with time will be assessed by adding a treatment x time interaction to the model and comparing models using a likelihood ratio test. Deaths will be accounted for by modelling survival and HRQoL jointly. Model fit will be assessed using standard methods and alternative models and/or transformations will be explored if appropriate. Treatment differences and 95% confidence intervals will be reported.

Missing data on patient questionnaires will be dealt with according to the scoring manuals. Multiple imputation methods will be used if greater than 5% of cases have missing data, otherwise complete case analysis will be undertaken. Compliance rates will be reported, including the number of participants who have withdrawn from the study, have been lost to follow-up or died. Causes of death for trial participants will be recorded.

Frequencies of adverse events will be described. The proportion of participants experiencing one or more serious adverse events in the two-year follow-up period will be compared using a generalised linear model.

For peer review only - http://bmjopen.bmj.com/site/about/guidelines.xhtml

Two subgroup analyses are planned: i) comparing primary and secondary outcomes by the experience level of the surgical site; and ii) comparing the primary outcome by type of mesothelioma (epithelioid, sarcomatoid or biphasic). An exploratory analysis investigating the effect of surgeon (surgical group only) will be performed for the primary outcome.

No interim analyses are planned. The primary analysis will take place when follow-up is complete for all recruited participants.

Economic evaluation

The economic evaluation will compare the costs and effects of surgery versus no surgery, following established guidelines as set out by NICE.[20] The within-trial cost-effectiveness analysis will be undertaken from an NHS and personal social services perspective, with a time horizon from time of consent to 24 months post-randomisation. The primary outcome measure for the economic evaluation will be quality adjusted life years (QALYs), estimated using the EuroQol EQ-5D-5L at each follow up timepoint.[15 16] Resource use data collection will be integrated into the trial CRFs for chemotherapy cycles and surgery (if applicable, this will include details of the surgical procedure, length of stay in hospital by level of care, and post-operative complications) and be collected at each follow up timepoint.

Unit costs will be sought to value resource use data, and the total costs per participant calculated. Responses to the EQ-5D-5L will be assigned valuations according to NICE guidance at the time of analysis,[21] and combined with survival to calculate QALYs gained per participant. Missing resource use and EQ-5D-5L data will be handled using multiple imputation methods.[22] From the average costs and QALYs gained in each trial group, the incremental cost-effectiveness ratio will be derived, producing an incremental cost per QALY gained of surgery compared to no surgery. Sensitivity analyses will assess the impact of varying key parameters on baseline cost-effectiveness results. Results will be expressed in terms of a cost-effectiveness acceptability curve, which indicates the likelihood that surgery is cost-effective for different levels of willingness to pay for health gain.

Ethics and dissemination

The study intervention is already routinely used in the NHS. This study has been reviewed and given favourable opinion by the London - Camberwell St. Giles Research Ethics Committee (REC; reference 13/LO/1481) on 7th November 2013. The pilot study was managed by Papworth Trials Unit Collaboration and the main trial is managed by the Bristol Trials Centre Clinical Trials and Evaluation Unit and sponsored by Royal Brompton &

For peer review only - http://bmjopen.bmj.com/site/about/guidelines.xhtml

Harefield NHS Foundation Trust. Each participant has the right to withdraw at any time. In addition, the investigator may withdraw the participant from their allocated treatment group if a clinical reason for not performing the surgical intervention is discovered. If a participant wishes to withdraw, any data already collected will be included in the study analyses, unless the participant expresses a wish for their data to be excluded. Withdrawing patients will be asked if they would continue in follow up and complete the requisite questionnaires. Participants who choose to withdraw from the study will be treated according to their hospitals' standard procedures.

The findings will be disseminated by usual academic channels, i.e. presentation at international meetings and peer-reviewed publications. A full report for the funder will be written on completion of the study and a lay summary of the results provided to patients.

Major changes to protocol

Since the first study protocol was approved by the REC (the current version is v6.0, 10 April 2019), the following changes have been made:

- Qualitative assessment sub-study added, as part of the pilot phase only.
- The EuroQol EQ-5D-5L was added.
- Updates to transition from pilot phase to main study, including addition of the integrated QRI and economic evaluation, and removal of the collection of blood and tissue samples, and one of the disease specific questionnaires – the EORTC QLQ LC-13.
- Length of follow up extended from two years until the end of the study for all participants to ensure that the study has 80% power.
- Video-recording aspect of the surgical quality assurance removed as this was deemed impractical by sites, and it was agreed that it was unnecessary by the Data Safety and Monitoring Committee and the Trial Steering Committee, acknowledging the other surgical quality assurance measures that are in place.

The relevant regulatory approvals were obtained for amendments to the protocol. Relevant parties (eg, investigators, trial participants) were informed.

Study progress

For peer review only - http://bmjopen.bmj.com/site/about/guidelines.xhtml

Recruitment started in May 2015 and 304 patients have been randomised so far (correct on 25 March 2020). 66 patients from the pilot study are included in this figure. Recruitment will continue until 1st June 2020.

The full protocol is available from:
https://www.journalslibrary.nihr.ac.uk/programmes/hta/1518831/

ADDITIONAL FIGURES

Figure 1. Trial schema showing the recruitment pathway for the MARS 2 study

ABBREVIATIONS

CONSORT- Consolidated Standards of Reporting Trials
CRF – Case Report Form
CT- computed tomography
ECOG- Eastern Cooperative Oncology Group
EORTC- European Organisation for Research and Treatment of Cancer
FEV1- forced expiratory volume after one second
HR- hazard ratio
HRQoL- health related quality of life
ISRCTN - International Standard Randomised Controlled Trials Number
MDT – multidisciplinary team
NHS- National Health Service
NICE- National Institute for Health and Care Excellence
NIHR- National Institute for Health Research
NYHA – New York Heart Association
PPI- patient and public involvement
QALY- quality adjusted life years
QLQ- quality of life questionnaire
QuinteT – Qualitative research integrated within trials
QRI- QuinteT Recruitment Intervention
RCT- randomised controlled trial
REC- Research Ethics Committee
TLco- transfer factor of the lung for carbon monoxide
TMG – Trial Management Group
UK- United Kingdom

Acknowledgements
The MARS 2 trial is sponsored by The Royal Brompton and Harefield NHS Foundation Trust. The sponsor will be responsible for the oversight of the MARS 2 study and to ensure the trial is managed appropriately. We want to thank the large teams at each hospital who work on the MARS 2 study (representatives from each are listed below). We would also like to thank Dr Fiona McDonald from the Royal Marsden Hospital, Dr Nagmi Qureshi from Papworth Hospital and Professor Simon Padley from the Royal Brompton Hospital for their radiotherapy advisory roles. Thank you also to Professor Andrew Nicholson for his histopathology advisory role for MARS 2.

For peer review only - http://bmjopen.bmj.com/site/about/guidelines.xhtml

Collaborators
***MARS 2 Trialists**
Project management team members:
Professor Eric Lim, Chief Investigator
Professor Chris Rogers, Methodological Lead and Statistician
Kate Ashton, Clinical Trial Coordinator
Athanasia Gravani, Clinical Trial Coordinator
Holly McKeon, Clinical Trial Coordinator
Wendy Underwood, Clinical Trial Coordinator
Dr Barbara Warnes, Clinical Trial Coordinator
Katherine Joyce, Assistant Clinical Trial Coordinator
Rachel Brophy, Assistant Clinical Trial Coordinator
Rosie Harris, Medical Statistician
Dr Nicola Mills, Senior Research Fellow, Quintet Research Intervention
Dr Daisy Elliott, Research Fellow, Quintet Research Intervention
Nicola Farrar, PhD Student, Quintet Research Intervention
Dr Elizabeth Stokes, Health Economist

Papworth Trials Unit Collaboration (pilot study):
Robert Rintoul, Director of Papworth Trials Unit Collaboration and Honorary Consultant Respiratory Physician
Victoria Hughes, Senior R&D Manager
Jane Elliott, Clinical Project Manager
Claire Matthews, Trial Manager
Philip Noyes, Trial Administrator

Participating Sites Members: pilot study and main trial
University Hospitals of Leicester NHS Trust – *medical and surgical site (opened 22/04/2015)*
Dean Fennell, Principal Investigator, Chair of Thoracic Medical Oncology
Apostolos Nakas, Consultant Thoracic Surgeon
Louise Nelson, Research Nurse
Sheffield Teaching Hospitals NHS Foundation Trust *– medical and surgical site (opened 13/05/2015)*
John Edwards, Principal Investigator, Consultant Thoracic Surgeon
Sara Tenconi, Consultant Thoracic Surgeon
Laura Socci, Consultant Thoracic Surgeon
Hilary Wood, Data Manager
Helena Hanratty, Research Nurse
Helena Stanley, Mesothelioma UK Clinical Nurse Specialist
South Tyneside and Sunderland NHS Foundation Trust *– medical site (opened 19/06/2015)*
Liz Fuller, Principal Investigator, Consultant Respiratory Physician
Judith Moore, Clinical Trials Officer
Papworth Hospital NHS Foundation Trust *– medical site (opened 01/07/2015)*
Robert Rintoul, Principal Investigator, Honorary Consultant Respiratory Physician
Suzanne Miller, Clinical Trials Coordinator
Amy Gladwell, Clinical Trials Administrator

For peer review only - http://bmjopen.bmj.com/site/about/guidelines.xhtml

Jenny Castedo, Research Nurse
Amanda Stone, Senior Research Nurse
Colchester Hospital University NHS Foundation Trust *– medical site (opened 03/11/2015)*
Dakshinamoorthy Muthukumar, Principal Investigator, Consultant Oncologist
Charlotte Ingle, Co-Investigator, Consultant Clinical Oncologist
Hayley Hewer, Research Nurse
South Tees Hospitals NHS Foundation Trust *– medical site (opened 16/11/2015)*
Talal Mansy, Principal Investigator, Consultant Medical Oncologist
Louise Li, Co-Investigator, Consultant Medical Oncologist
Eleanor Aynsley, Co-Investigator, Consultant Clinical Oncologist
Andrea Watson, Clinical Trials Coordinator
Charlotte Jacobs, Clinical Trials Coordinator
The Clatterbridge Cancer Centre NHS Foundation Trust *– medical site (opened 25/11/2015)*
Tony Pope, Principal Investigator, Consultant in Clinical Oncology
Alison Hassall, Research Nurse
Masuma Begum, Clinical Trials Assistant
University Hospitals of Derby and Burton NHS Foundation Trust *– medical site, (opened 26/11/2015)*
Manjusha Keni, Principal Investigator, Consultant Clinical Oncologist
Christopher Worth, Cancer Clinical Research Nurse Lead
Ellie Piggott, Research Practitioner
Elizabeth Nadin, Research Practitioner
Leeds Teaching Hospitals NHS Trust – *medical site (opened 02/12/2015)*
Richard Milton, Principal Investigator, Consultant Thoracic Surgeon
Victoria Ashford-Turner, Research Nurse
Matthew Callister, Respiratory Consultant
Manchester University NHS Foundation Trust *– medical site (opened 21/12/2015)*
Paul Taylor, Principal Investigator
Yvonne Summers, Sub Investigator
Raffaele Califano, Sub Investigator
Laura Cove-Smith, Sub Investigator
Matt Evison, Sub Investigator
Maria Blinston, Research Nurse
Sara Waplington, Clinical Trials Coordinator
Amal Ismail, Clinical Trials Administrator
Rachel Chant, Research Nurse
Asmita Desai, Research Nurse
Juliette Novasio, Clinical Trials Manager
Marie Kirwan, Research Nurse
The Royal Wolverhampton NHS Trust *– medical site (opened 04/01/2016)*
Pek Koh, Principal Investigator, Consultant Clinical Oncologist
Ian Morgan, previous Principal Investigator, Consultant Cardiothoracic Surgeon
Victoria Lake, Research Nurse
Nichola Harris, Research Nurse
Royal Gwent Hospital, Aneurin Bevan University Health Board *– medical site (opened 08/02/2016)*

For peer review only - http://bmjopen.bmj.com/site/about/guidelines.xhtml

Andreea Alina Ionescu, Principal Investigator, Consultant Respiratory Physician
Simon Hodge, Research Nurse

The Royal Marsden NHS Foundation Trust *– medical site (opened 08/04/2016)*

Prof Sanjay Popat, Principal Investigator, Consultant Medical Oncologist

Chelsea sub-site

Dr Nadia Yousaf, Sub-Investigator
Dr Nadza Tokaca, Sub-Investigator
Dr Adam Januszewski, Sub-Investigator
Dr Avani Athauda, Sub-Investigator
Dr Anisha Ramessur, Sub-Investigator
Dr Emily Grist, Sub-Investigator
Dr Niamh Colman, Sub-Investigator
Dr Michael Flynn, Sub-Investigator
Joan Joyce, Research Nurse
Sarah Vaughan, Research Nurse
Maria Piga, Senior Research Nurse
Derya Sahin, Senior Research Nurse
Agnieszka Yongue, Senior Trials Coordinator
Emma Turay, Clinical Trials Administrator

Sutton sub-site

Prof Mary O'Brien, Sub-Investigator
Dr Jaishree Bhosle, Sub-Investigator
Dr Rajiv Kumar, Sub-Investigator
Dr Charlotte Milner-Watts, Sub-Investigator
Dr Jessica Brown, Sub-Investigator
Dr David Walder, Sub-Investigator
Dr Alexandros Georgiou, Sub-Investigator
Dr Xiaorong Wu, Sub-Investigator
Dr Naila Kaudeer, Sub-Investigator
Dr Kroopa Joshi, Sub-Investigator
Dr Michael Davidson, Sub-Investigator
Dr Shelize Khakoo, Sub-Investigator
Bee Ayite, Research Nurse
Kathryn Priest, Research Nurse
James Dobbyn, Senior Research Nurse
Vasanthi Prathapan, Senior Research Nurse
Deborah McCrimmon, Research Radiographer
Natalie Ash, Assistant Practitioner
Alison Norton, Senior Clinical Trial Coordinator
Bianca Peet, Senior Trials Manager
Libby Hennessy, Clinical Trials Administrator
Rosemary Johnson, Clinical Trials Administrator
Laura White, Clinical Trials Administrator

Kingston sub-site

Dr Edward Armstrong, Sub-Investigator
Dr Maria Coakley, Sub-Investigator
Dr Scott Shepherd, Sub-Investigator
Dr Narda Chaabouni, Sub-Investigator

For peer review only - http://bmjopen.bmj.com/site/about/guidelines.xhtml

Dr Katherina Sreter, Sub-Investigator
Dr Vasileios Angelis, Sub-Investigator
Dr Mariko Morishita, Sub-Investigator
Dr Jose Roca, Sub-Investigator
Mary Jane de los Reyes Lauigan, Research Nurse
Katrin Sainudeen, Clinical Trials Practitioner
Helen Morgan, Clinical Trials Administrator
Peterborough City Hospital, North West Anglia NHS Foundation Trust *– medical site (opened 16/05/2016)*
Dr Sarah Treece, Principal Investigator, Consultant Clinical Oncologist
Dr Abigail Hollingdale, Co-Investigator, Consultant Clinical Oncologist
Chloe Eddings, Study Co-ordinator/Research Nurse
Holly Warman, Study Co-ordinator/Clinical Trials Assistant

Participating Sites Members: main trial only
Barts Health NHS Trust *– medical and surgical site (opened 05/06/2017)*
Kelvin Lau, Principal Investigator, Consultant Thoracic Surgeon
David Waller, Consultant Thoracic Surgeon
Jeremy Steele, Consultant Medical Oncologist
Jo Hargrave, Mesothelioma Clinical Nurse Specialist
Resmi Jayachandran, Senior Clinical Trials Practitioner
Pratistha Panday, Clinical Trials Assistant
The Beatson West of Scotland Cancer Centre, Greater Glasgow Health Board *– medical site (opened 14/07/2017)*
Clinton Ali, Principal Investigator, Consultant Medical Oncologist
Austin McInnes, Clinical Trial Coordinator
Golden Jubilee National Hospital *– surgical site (opened 14/07/2017)*
Alan Kirk, Principal Investigator, Consultant Thoracic Surgeon
Rocco Bilancia, Consultant Thoracic Surgeon
Julie Buckley, Research Nurse
Elizabeth Boyd, Research Nurse
North Bristol NHS Trust *– medical site (opened 28/02/2018)*
Nick Maskell, Principal Investigator, Consultant in Respiratory Medicine
Natalie Zahan-Evans, Research Nurse
Anna Morley, Senior Research Nurse
Norfolk and Norwich University Hospitals NHS Foundation Trust *– medical site (opened 12/06/2018)*
Zacharias Tasigiannopoulos, Principal Investigator, Consultant Clinical Oncologist
Adela Dann, Clinical Trials Practitioner
Eleanor Mishra, Consultant Respiratory Physician
Pinelopi Gkogkou, Consultant Clinical Oncologist
University Hospitals Plymouth NHS Trust *– medical site (opened 16/07/2018)*
Amy Roy, Principal Investigator, Consultant Clinical Oncologist
Irene Harvey, Research Nurse
Hilary Congdon, Research Nurse
Barking, Havering and Redbridge University Hospitals NHS Trust *– medical site (opened 24/07/2018)*
Jonathan Shamash, Principal Investigator, Consultant Medical Oncologist

For peer review only - http://bmjopen.bmj.com/site/about/guidelines.xhtml

Alison Ray, Clinical Trials Nurse
Guy's and St. Thomas' NHS Foundation Trust *– medical and surgical site (opened 10/08/2018)*
Andrea Bille, Principal Investigator, Consultant Thoracic Surgeon
Jehan Mansi, Clinical Trials Practitioner
Amy Quinn, Clinical Research Nurse
Oxford University Hospitals NHS Foundation Trust *– medical site (opened 19/10/2018)*
Najib M Rahman, Principal Investigator, Consultant and Senior Lecturer in Respiratory Medicine
Jack Seymour, Research Nurse
Hannah Ball, Mesothelioma Nurse
Meenali Chitnis, Consultant Clinical Oncologist
Maidstone and Tunbridge Wells NHS Trust *– medical site (opened 19/10/2018)*
Riyaz Shah, Principal Investigator, Consultant Medical Oncologist
Eirini Petroyannou, Research Nurse
Kimberley Snoad, Clinical Trials Administrator
Monica Tavares Barbosa, Research Nurse
University Hospitals Birmingham NHS Foundation Trust *– medical site (opened 03/01/2019)*
Professor Gary Middleton, Principal Investigator, Professor in Medical Oncology
Philip Earwaker, Consultant Medical Oncologist
Haider Abbas, Consultant Medical Oncologist
Parminder Sohal, Oncology Research Sister

Independent Trial Steering Committee members

Prof Marcus Flather MBBS FRCP (Chair), Clinical Professor in Medicine; Dr Paul Beckett, Consultant Respiratory Physician; Miss Carol Tan, Consultant Thoracic Surgeon - has declared the following competing interest: ethicon endostaplers – consultancies; Prof Fergus Gleeson FRCP FRCR, Professor of Radiology and Consultant Radiologist; Prof Fergus MacBeth, Consultant Oncologist; Hon Dr Mavis Nye, Patient Advocate; Dr Harvey Pass, Professor of Thoracic Oncology; Dr Pauline Leonard, Consultant Medical Oncologist – has declared the following competing interests: Teva, Amgen, LCO Pharma; Prof Tom Treasure MS MD FRCS FRCP, Clinical Operations Research Unit.
Unless otherwise stated above, committee members have declared no competing interests.

Independent Data Monitoring and Safety Committee members

Prof Linda Sharples (Chair), Professor of Medical Statistics; Prof Valerie Rusch, Consultant Thoracic Surgeon; Prof Mark Britton, Consultant Physician; Dr Robin Rudd, London Lung Cancer Group; Prof Joseph S. Friedberg MD FACS, Professor of Surgery and Surgeon-in-Chief; Prof Peter Goldstraw, Emeritus Professor of Thoracic Surgery.
Unless otherwise stated above, committee members have declared no competing interests.

Contributors

EL - Study design, preparation and drafting of protocol and manuscript, Chief Investigator for the trial; LD - Study design, preparation of protocol and review of manuscript; JGE - Study design, preparation of protocol and review of manuscript, Principal Investigator and acquired data for the study; DE - Design of integrated qualitative study, preparation of study protocol, review of manuscript; DAF - Study design, preparation of protocol and review of manuscript,

For peer review only - http://bmjopen.bmj.com/site/about/guidelines.xhtml

Principal Investigator and acquired data for the study; SP - Study design, preparation of protocol and review of manuscript, Principal Investigator and acquired data for the study; RR - Study design, preparation of protocol and review of manuscript, Principal Investigator and acquired data for the study; DW - Study design, preparation of protocol and review of manuscript, acquired data for the study; CA - Review of manuscript, Principal Investigator and acquired data for the study; AB - Review of manuscript, Principal Investigator and acquired data for the study; LF - Review of manuscript, Principal Investigator and acquired data for the study; AAI - Review of manuscript, Principal Investigator and acquired data for the study; MK - Review of manuscript, Principal Investigator and acquired data for the study; AK - Review of manuscript, Principal Investigator and acquired data for the study; PK - Review of manuscript, Principal Investigator and acquired data for the study; KL - Review of manuscript, Principal Investigator and acquired data for the study; TM - Review of manuscript, Principal Investigator and acquired data for the study; NAM - Review of manuscript, Principal Investigator and acquired data for the study; RM - Review of manuscript, Principal Investigator and acquired data for the study; DM - Review of manuscript, Principal Investigator and acquired data for the study; TP- Review of manuscript, Principal Investigator and acquired data for the study; AR - Review of manuscript, Principal Investigator and acquired data for the study; RS - Review of manuscript, Principal Investigator and acquired data for the study; JS - Review of manuscript, Principal Investigator and acquired data for the study; ZT - Review of manuscript, Principal Investigator and acquired data for the study; PT - Review of manuscript, Principal Investigator and acquired data for the study; ST - Review of manuscript, Principal Investigator and acquired data for the study; KA - Study design, preparation and drafting of protocol and manuscript, oversaw study conduct and acquisition of data; RAH - Statistical analysis plan, review of manuscript; KJ - Preparation and drafting of manuscript, oversaw study conduct and acquisition of data; BW- Drafting of manuscript, oversaw study conduct and acquisition of data; NM - Conduct of integrated qualitative study, preparation of study protocol, review of manuscript; ES - Design of health economic component, preparation of study protocol, review of manuscript; CAR - Study design, sample size and statistical analysis plan, drafting of protocol, review of manuscript.

Funding

This research is funded by the National Institute for Health Research (NIHR) Health Technology Assessment (HTA) Programme (project number 15/188/31). The pilot study was funded by Cancer Research UK and Mesothelioma UK has contributed towards patient travel expenses.

This study was designed and delivered in collaboration with the Bristol Trials Centre Clinical Trials and Evaluation Unit (CTEU), a UK Clinical Research Collaboration (UKCRC) registered clinical trials unit which, as part of the Bristol Trials Centre (BTC), is in receipt of NIHR Clinical Trials Unit (CTU) support funding.

Daisy Elliott was supported by the NIHR Biomedical Research Centre at University Hospitals Bristol NHS Foundation Trust and the University of Bristol. The views expressed in this publication are those of the author(s) and not necessarily those of the NHS, the National Institute for Health Research or the Department of Health and Social Care.
Sanjay Popat acknowledges NHS funding to the Royal Marsden Hospital / Institute of Cancer Research NIHR Biomedical Research Centre.

For peer review only - http://bmjopen.bmj.com/site/about/guidelines.xhtml

Disclaimer

The views and opinions expressed therein are those of the authors and do not necessarily reflect those of the HTA programme, NIHR, NHS or the Department of Health and Social Care.

The funder and sponsor approve any amendments to the study but have no direct involvement in study design; collection, management, analysis, and interpretation of data; writing of the report; and the decision to Submit this report for publication.

Competing interests' statement

EL: reports personal fees from Abbott Molecular, personal fees from Glaxo Smith Kline, personal fees from Pfizer, personal fees from Norvatis, personal fees from Covidien, personal fees from Roche, personal fees from Lily Oncology, personal fees from Boehringer Ingelheim, personal fees from Medela, grants and personal fees from ScreenCell, personal fees from Ethicon, grants from Clearbridge Biomedics, grants from Illumina, grants from Guardant Health, personal fees from AstraZenecia (outside the submitted work), a patent P52435GB issued to Imperial Innovations, and a patent P57988GB issued to Imperial Innovations and Director of lung screening at Cromwell Hospital, CI for VIOLET NIHR HTA (13/04/03), CI for MARS 2 NIHR HTA (15/188/31); DAF: reports grants from Astex Therapeutics, personal fees from Aldeyra, grants from Boehringer Ingelheim, non-financial support from Clovis, non-financial support from Eli Lilly, from BMS, personal fees from Inventiva, personal fees from Paredox, personal fees and non-financial support from Roche, grants from MSD, grants from Bayer, during the conduct of the study; SP: reports personal fees from BMS, personal fees from Roche, personal fees from Takeda, personal fees from AstraZeneca, personal fees from Pfizer, personal fees from MSD, personal fees from EMD Serono, personal fees from Guardant Health, personal fees from Abbvie, personal fees from Boehringer Ingelheim, personal fees from OncLive, personal fees from Medscape, personal fees from Incyte (outside the submitted work); PK: reports non-Financial Support: Travel grant to ESMO Congress 2018, was sponsored by Boeringer Ingelheim, West Midlands UK Oncology Specialist Trainee Regional Training Day April 2018, meeting was sponsored by Roche, Servier and Bristol Myers Squibb in the purchase of exhibition stand space, Eisai was sponsoring this meeting towards the cost of catering. TM: reports other from MSD, other from Roche, other from Tesaro, from AstraZeneca, personal fees from Tesaro, personal fees from Roche, personal fees from MSD, personal fees from Amgen, other from BMS (outside the submitted work); KA: reports grants from NIHR HTA, during the conduct of the study; RAH: reports grants from NIHR HTA, during the conduct of the study; KJ: reports grants from NIHR HTA, during the conduct of the study; ES: reports grants from UK National Institute for Health Research (Health Technology Assessment Programme), Department of Health and Social Care, during the conduct of the study; CAR: reports grants from National Institute for Health Research, during the conduct of the study.

Unless otherwise stated above, authors have declared no competing interests.

Patient Consent for publication

Not required.

Ethics Approval

For peer review only

This study received a favourable opinion by the London - Camberwell St. Giles Research Ethics Committee (REC; reference 13/LO/1481) on 7th November 2013.

Provenance and peer review
Not commissioned, externally peer reviewed.

Data sharing
Data will not be made available for sharing until after publication of the main results of the study. Thereafter, anonymised individual patient data will be made available for secondary research, conditional on prior consent having been given by the patients and assurance from the secondary researcher that the proposed use of the data is compliant with the Medical Research Council (MRC) Policy on Data Sharing regarding scientific quality, ethical requirements and value for money. A minimum requirement with respect to scientific quality will be a publicly available pre-specified protocol describing the purpose, methods and analysis of the secondary research, e.g. a protocol for a Cochrane systematic review. The second file containing patient identifiers would be retained for record linkage or a similar purpose, Subject to confirmation that the secondary research protocol has been approved by a UK REC or other similar, approved ethics review body.

ORCID IDs
Daisy Elliott: 0000-0001-8143-9549; Robert Rintoul: 0000-0003-3875-3780; Katherine Joyce: 0000-0002-5539-7178; Chris A Rogers: 0000-0002-9624-2615

Exclusive Licence

I, the Submitting Author has the right to grant and does grant on behalf of all authors of the Work (as defined in the below author licence), an exclusive licence and/or a non-exclusive licence for contributions from authors who are: i) UK Crown employees; ii) where BMJ has agreed a CC-BY licence shall apply, and/or iii) in accordance with the terms applicable for US Federal Government officers or employees acting as part of their official duties; on a worldwide, perpetual, irrevocable, royalty-free basis to BMJ Publishing Group Ltd ("BMJ") its licensees and where the relevant Journal is co-owned by BMJ to the co-owners of the Journal, to publish the Work in BMJ Open and any other BMJ products and to exploit all rights, as set out in our licence.

The Submitting Author accepts and understands that any supply made under these terms is made by BMJ to the Submitting Author unless you are acting as an employee on behalf of your employer or a postgraduate student of an affiliated institution which is paying any applicable article publishing charge ("APC") for Open Access articles. Where the Submitting Author wishes to make the Work available on an Open Access basis (and intends to pay the relevant APC), the terms of reuse of such Open Access shall be governed by a Creative Commons licence – details of these licences and which Creative Commons licence will apply to this Work are set out in our licence referred to above.

For peer review only - http://bmjopen.bmj.com/site/about/guidelines.xhtml

References

1. Executive HaS. Asbestos-related disease statistics in Great Britain, 2019 2019.
2. Peto J, Hodgson JT, Matthews FE, et al. Continuing increase in mesothelioma mortality in Britain. *Lancet* 1995;345(8949):535-9.
3. National Institute of Health and Care Excellence. Pemetrexed for the treatment of malignant pleural mesothelioma. Technology appraisal guidance [TA135]. 2008
4. Vogelzang NJ, Rusthoven JJ, Symanowski J, et al. Phase III study of pemetrexed in combination with cisplatin versus cisplatin alone in patients with malignant pleural mesothelioma. *Journal of clinical oncology* 2003;21(14):2636-44.
5. Royal College of Physicians. National Lung Cancer Audit pleural mesothelioma report 2016 (for the audit period 2014). London: Royal College of Physicians, 2016.
6. Rice D, Rusch V, Pass H, et al. Recommendations for uniform definitions of surgical techniques for malignant pleural mesothelioma: a consensus report of the international association for the study of lung cancer international staging committee and the international mesothelioma interest group. *Journal of thoracic oncology* 2011;6(8):1304-12.
7. Treasure T, Lang-Lazdunski L, Waller D, et al. Extra-pleural pneumonectomy versus no extra-pleural pneumonectomy for patients with malignant pleural mesothelioma: clinical outcomes of the Mesothelioma and Radical Surgery (MARS) randomised feasibility study. *The Lancet Oncology* 2011;12(8):763-72. doi: 10.1016/S1470-2045(11)70149-8
8. Rintoul RC, Ritchie AJ, Edwards JG, et al. Efficacy and cost of video-assisted thoracoscopic partial pleurectomy versus talc pleurodesis in patients with malignant pleural mesothelioma (MesoVATS): an open-label, randomised, controlled trial. *Lancet* 2014;384(9948):1118-27. doi: 10.1016/S0140-6736(14)60418-9
9. Cao C, Tian DH, Pataky KA, et al. Systematic review of pleurectomy in the treatment of malignant pleural mesothelioma. *Lung Cancer* 2013;81(3):319-27. doi: 10.1016/j.lungcan.2013.04.024
10. Lang-Lazdunski L, Bille A, Lal R, et al. Pleurectomy/decortication is superior to extrapleural pneumonectomy in the multimodality management of patients with malignant pleural mesothelioma. *Journal of Thoracic Oncology* 2012;7(4):737-43. doi: 10.1097/JTO.0b013e31824ab6c5
11. Teh E, Fiorentino F, Tan C, et al. A systematic review of lung-sparing extirpative surgery for pleural mesothelioma. *Journal of the Royal Society of Medicine* 2011;104(2):69-80. doi: 10.1258/jrsm.2010.100345
12. Donovan JL, Rooshenas L, Jepson M, et al. Optimising recruitment and informed consent in randomised controlled trials: the development and implementation of the Quintet Recruitment Intervention (QRI). *Trials* 2016;17(1):283. doi: 10.1186/s13063-016-1391-4
13. Rooshenas L, Paramasivan S, Jepson M, et al. Intensive Triangulation of Qualitative Research and Quantitative Data to Improve Recruitment to Randomized Trials: The QuinteT Approach. *Qual Health Res* 2019;29(5):672-79. doi: 10.1177/1049732319828693 [published Online First: 2019/02/23]
14. Rooshenas L, Scott LJ, Blazeby JM, et al. The QuinteT Recruitment Intervention supported five randomized trials to recruit to target: a mixed-methods evaluation. *J Clin Epidemiol* 2019;106:108-20. doi: 10.1016/j.jclinepi.2018.10.004 [published Online First: 2018/10/20]
15. EuroQol Group. EuroQol - a new facility for the measurement of health-related quality of life. *Health policy* 1990;16(3):199-208.
16. Herdman M, Gudex C, Lloyd A, et al. Development and preliminary testing of the new five-level version of EQ-5D (EQ-5D-5L). *Quality of Life Research* 2011;20(10):1727-36. doi: 10.1007/s11136-011-9903-x [published Online First: 2011/04/12]
17. Utley M, Fiorentino F, Treasure T. Obtaining an upper estimate of the survival benefit associated with surgery for mesothelioma. *European Journal of Cardiothoracic Surgery* 2010;38:241-4.

For peer review only - http://bmjopen.bmj.com/site/about/guidelines.xhtml

18. Briel M, Olu KK, von Elm E, et al. A systematic review of discontinued trials suggested that most reasons for recruitment failure were preventable. *Journal of Clinical Epidemiology* 2016;80:8-15. doi: 10.1016/j.jclinepi.2016.07.016 [published Online First: 2016/08/09]
19. Blencowe NS, Cook JA, Pinkney T, et al. Delivering successful randomized controlled trials in surgery: Methods to optimize collaboration and study design. *Clinical Trials* 2017;14(2):211-18. doi: 10.1177/1740774516687272 [published Online First: 2017/02/01]
20. National Institute for Health and Care Excellence. Guide to the Methods of Technology Appraisal 2013. London: National Institute for Health and Care Excellence, 2013.
21. National Institute for Health and Care Excellence. National Institute for Health and Care Excellence. Position statement on use of the EQ-5D-5L valuation set for England (updated October 2019) [updated October 2019. Available from: https://www.nice.org.uk/about/what-we-do/our-programmes/nice-guidance/technology-appraisal-guidance/eq-5d-5l accessed 18 December 2019.
22. White IR, Royston P, Wood AM. Multiple imputation using chained equations: Issues and guidance for practice. *Stat Med* 2011;30(4):377-99.

For peer review only

For peer review only - http://bmjopen.bmj.com/site/about/guidelines.xhtml

Figure 1 Trial schema showing the recruitment pathway for the MARS 2 study

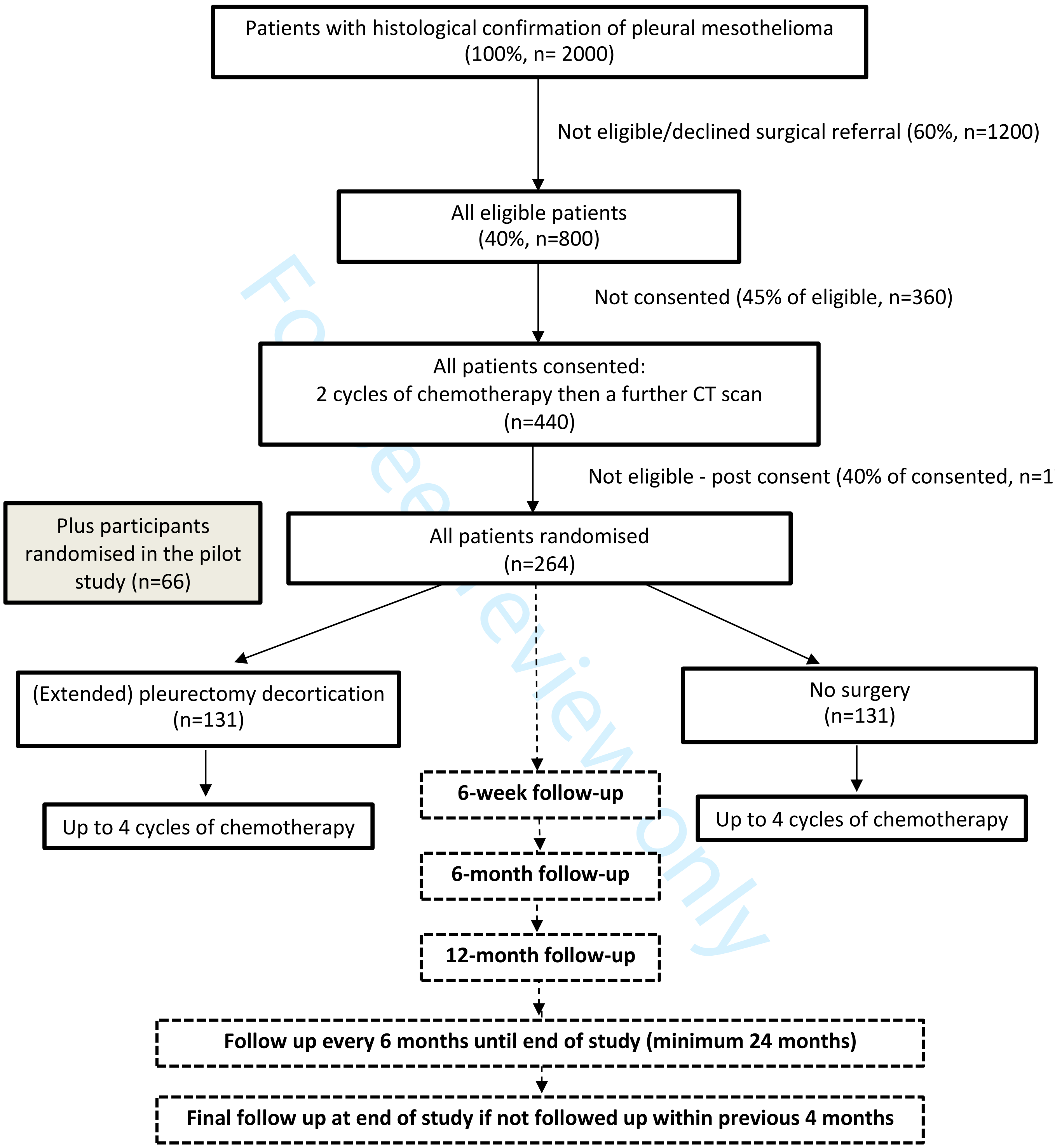

For peer review only

For peer review only - http://bmjopen.bmj.com/site/about/guidelines.xhtml

SPIRIT

STANDARD PROTOCOL ITEMS: RECOMMENDATIONS FOR INTERVENTIONAL TRIALS

SPIRIT 2013 Checklist: Recommended items to address in a clinical trial protocol and related documents*

Section/item	Item No	Description	Addressed on page number
**Administrative information**			
Title	1	Descriptive title identifying the study design, population, interventions, and, if applicable, trial acronym	1
Trial registration	2a	Trial identifier and registry name. If not yet registered, name of intended registry	4
	2b	All items from the World Health Organization Trial Registration Data Set	N/A
Protocol version	3	Date and version identifier	16
Funding	4	Sources and types of financial, material, and other support	23
Roles and responsibilities	5a	Names, affiliations, and roles of protocol contributors	1-3,23
	5b	Name and contact information for the trial sponsor	18
	5c	Role of study sponsor and funders, if any, in study design; collection, management, analysis, and interpretation of data; writing of the report; and the decision to submit the report for publication, including whether they will have ultimate authority over any of these activities	24
	5d	Composition, roles, and responsibilities of the coordinating centre, steering committee, endpoint adjudication committee, data management team, and other individuals or groups overseeing the trial, if applicable (see Item 21a for data monitoring committee)	N/A

For peer review only

**Introduction**			
Background and rationale	6a	Description of research question and justification for undertaking the trial, including summary of relevant studies (published and unpublished) examining benefits and harms for each intervention	5,6
	6b	Explanation for choice of comparators	6
Objectives	7	Specific objectives or hypotheses	6,7
Trial design	8	Description of trial design including type of trial (eg, parallel group, crossover, factorial, single group), allocation ratio, and framework (eg, superiority, equivalence, noninferiority, exploratory)	7
**Methods: Participants, interventions, and outcomes**			
Study setting	9	Description of study settings (eg, community clinic, academic hospital) and list of countries where data will be collected. Reference to where list of study sites can be obtained	7,8
Eligibility criteria	10	Inclusion and exclusion criteria for participants. If applicable, eligibility criteria for study centres and individuals who will perform the interventions (eg, surgeons, psychotherapists)	8
Interventions	11a	Interventions for each group with sufficient detail to allow replication, including how and when they will be administered	8,9
	11b	Criteria for discontinuing or modifying allocated interventions for a given trial participant (eg, drug dose change in response to harms, participant request, or improving/worsening disease)	8,9
	11c	Strategies to improve adherence to intervention protocols, and any procedures for monitoring adherence (eg, drug tablet return, laboratory tests)	7,8, 13,14
	11d	Relevant concomitant care and interventions that are permitted or prohibited during the trial	9
Outcomes	12	Primary, secondary, and other outcomes, including the specific measurement variable (eg, systolic blood pressure), analysis metric (eg, change from baseline, final value, time to event), method of aggregation (eg, median, proportion), and time point for each outcome. Explanation of the clinical relevance of chosen efficacy and harm outcomes is strongly recommended	9-11
Participant timeline	13	Time schedule of enrolment, interventions (including any run-ins and washouts), assessments, and visits for participants. A schematic diagram is highly recommended (see Figure)	11

For peer review only - http://bmjopen.bmj.com/site/about/guidelines.xhtml

Sample size	14	Estimated number of participants needed to achieve study objectives and how it was determined, including clinical and statistical assumptions supporting any sample size calculations	12,13
Recruitment	15	Strategies for achieving adequate participant enrolment to reach target sample size	12-14
**Methods: Assignment of interventions (for controlled trials)**			
Allocation:			
Sequence generation	16a	Method of generating the allocation sequence (eg, computer-generated random numbers), and list of any factors for stratification. To reduce predictability of a random sequence, details of any planned restriction (eg, blocking) should be provided in a separate document that is unavailable to those who enrol participants or assign interventions	9
Allocation concealment mechanism	16b	Mechanism of implementing the allocation sequence (eg, central telephone; sequentially numbered, opaque, sealed envelopes), describing any steps to conceal the sequence until interventions are assigned	9
Implementation	16c	Who will generate the allocation sequence, who will enrol participants, and who will assign participants to interventions	9
Blinding (masking)	17a	Who will be blinded after assignment to interventions (eg, trial participants, care providers, outcome assessors, data analysts), and how	11
	17b	If blinded, circumstances under which unblinding is permissible, and procedure for revealing a participant's allocated intervention during the trial	N/A
**Methods: Data collection, management, and analysis**			
Data collection methods	18a	Plans for assessment and collection of outcome, baseline, and other trial data, including any related processes to promote data quality (eg, duplicate measurements, training of assessors) and a description of study instruments (eg, questionnaires, laboratory tests) along with their reliability and validity, if known. Reference to where data collection forms can be found, if not in the protocol	10,11,12
	18b	Plans to promote participant retention and complete follow-up, including list of any outcome data to be collected for participants who discontinue or deviate from intervention protocols	14-16

For peer review only - http://bmjopen.bmj.com/site/about/guidelines.xhtml

Data management	19	Plans for data entry, coding, security, and storage, including any related processes to promote data quality (eg, double data entry; range checks for data values). Reference to where details of data management procedures can be found, if not in the protocol	N/A
Statistical methods	20a	Statistical methods for analysing primary and secondary outcomes. Reference to where other details of the statistical analysis plan can be found, if not in the protocol	14,15
	20b	Methods for any additional analyses (eg, subgroup and adjusted analyses)	15
	20c	Definition of analysis population relating to protocol non-adherence (eg, as randomised analysis), and any statistical methods to handle missing data (eg, multiple imputation)	14
**Methods: Monitoring**			
Data monitoring	21a	Composition of data monitoring committee (DMC); summary of its role and reporting structure; statement of whether it is independent from the sponsor and competing interests; and reference to where further details about its charter can be found, if not in the protocol. Alternatively, an explanation of why a DMC is not needed	22
	21b	Description of any interim analyses and stopping guidelines, including who will have access to these interim results and make the final decision to terminate the trial	N/A
Harms	22	Plans for collecting, assessing, reporting, and managing solicited and spontaneously reported adverse events and other unintended effects of trial interventions or trial conduct	N/A
Auditing	23	Frequency and procedures for auditing trial conduct, if any, and whether the process will be independent from investigators and the sponsor	N/A
**Ethics and dissemination**			
Research ethics approval	24	Plans for seeking research ethics committee/institutional review board (REC/IRB) approval	15,16
Protocol amendments	25	Plans for communicating important protocol modifications (eg, changes to eligibility criteria, outcomes, analyses) to relevant parties (eg, investigators, REC/IRBs, trial participants, trial registries, journals, regulators)	16

Consent or assent	26a	Who will obtain informed consent or assent from potential trial participants or authorised surrogates, and how (see Item 32)	9
	26b	Additional consent provisions for collection and use of participant data and biological specimens in ancillary studies, if applicable	N/A
Confidentiality	27	How personal information about potential and enrolled participants will be collected, shared, and maintained in order to protect confidentiality before, during, and after the trial	25
Declaration of interests	28	Financial and other competing interests for principal investigators for the overall trial and each study site	23, 24
Access to data	29	Statement of who will have access to the final trial dataset, and disclosure of contractual agreements that limit such access for investigators	25
Ancillary and post-trial care	30	Provisions, if any, for ancillary and post-trial care, and for compensation to those who suffer harm from trial participation	N/A
Dissemination policy	31a	Plans for investigators and sponsor to communicate trial results to participants, healthcare professionals, the public, and other relevant groups (eg, via publication, reporting in results databases, or other data sharing arrangements), including any publication restrictions	4, 16
	31b	Authorship eligibility guidelines and any intended use of professional writers	N/A
	31c	Plans, if any, for granting public access to the full protocol, participant-level dataset, and statistical code	N/A
**Appendices**			
Informed consent materials	32	Model consent form and other related documentation given to participants and authorised surrogates	On request
Biological specimens	33	Plans for collection, laboratory evaluation, and storage of biological specimens for genetic or molecular analysis in the current trial and for future use in ancillary studies, if applicable	N/A

*It is strongly recommended that this checklist be read in conjunction with the SPIRIT 2013 Explanation & Elaboration for important clarification on the items. Amendments to the protocol should be tracked and dated. The SPIRIT checklist is copyrighted by the SPIRIT Group under the Creative Commons "Attribution-NonCommercial-NoDerivs 3.0 Unported" license.

For peer review only

BMJ Open

Mesothelioma and Radical Surgery 2 (MARS 2): protocol for a multicentre randomised trial comparing (extended) pleurectomy decortication versus no (extended) pleurectomy decortication for patients with malignant pleural mesothelioma

Journal:	*BMJ Open*
Manuscript ID	bmjopen-2020-038892.R1
Article Type:	Protocol
Date Submitted by the Author:	27-May-2020

Complete List of Authors:	Lim, Eric; Royal Brompton and Harefield NHS Trust, Academic Division of Thoracic Surgery<br>Darlison, Liz; University Hospitals of Leicester NHS Trust, The Glenfield Hospital; Mesothelioma UK, c/o Glenfield Hospital<br>Edwards , John ; Sheffield Teaching Hospitals NHS Foundation Trust, Cardiothoracic Centre<br>Elliott, Daisy; University of Bristol, Population Health Sciences<br>Fennell, DA; University Hospitals of Leicester NHS Trust,<br>Popat, Sanjay; Royal Marsden NHS Foundation Trust, Department of Medicine<br>Rintoul, Robert; Papworth Hospital NHS Foundation Trust, Department of Thoracic Oncology<br>Waller , David ; Barts Health NHS Trust, Department of Thoracic Surgery<br>Ali, Clinton ; Gartnavel General Hospital, The Beatson West of Scotland Cancer Centre<br>Bille, Andrea; Guy's and Saint Thomas' Hospitals NHS Trust, Thoracic Surgery<br>Fuller , Liz; South Tyneside NHS Foundation Trust, Respiratory Medicine<br>Ionescu, Andreea; Aneurin Bevan Health Board, Lung Cancer<br>Keni, Manjusha; Derby Teaching Hospitals NHS Foundation Trust, Oncology<br>Kirk , Alan ; Golden Jubilee National Hospital, Department of Thoracic Surgery<br>Koh, Pek; New Cross Hospital, Department of Oncology<br>Lau, Kelvin ; Barts Health NHS Trust, Department of Thoracic Surgery<br>Mansy, Talal; South Tees Hospitals NHS Foundation Trust, Oncology<br>Maskell, Nick; University of Bristol, Academic Respiratory Unit, School of Clinical Sciences; North Bristol NHS Trust, Respiratory Research Unit<br>Milton , Richard ; Leeds Teaching Hospitals NHS Trust, Thoracic Surgery Department<br>Muthukumar, Dakshinamoorthy; Colchester Hospital University NHS Foundation Trust, Oncology<br>Pope , Tony ; Clatterbridge Cancer Centre NHS Foundation Trust, Clatterbridge Cancer Centre<br>Roy , Amy ; University Hospitals Plymouth NHS Trust, Plymouth Oncology Centre<br>Shah, Riyaz; Maidstone and Tunbridge Wells NHS Trust

	Shamash , Jonathan; Barts Health NHS Trust, Department of Medical Oncology<br>Tasigiannopoulos, Zacharias ; Norfolk and Norwich University Hospital NHS Trust, The Oncology Care Team<br>Taylor, Paul; The Christie NHS Foundation Trust, Medical Oncology<br>Treece , Sarah; North West Anglia NHS Foundation Trust, Heamatology and Oncology Unit<br>Ashton, Kate; University of Bristol, Bristol Trials Centre (CTEU)<br>Harris, Rosie; University of Bristol, Bristol Trials Centre (CTEU)<br>Joyce, Katherine; University of Bristol, Bristol Trials Centre (CTEU)<br>Warnes, Barbara; University of Bristol, Bristol Trial Centre (CTEU)<br>Mills, Nicola; University of Bristol, Population Health Sciences<br>Stokes, Elizabeth; University of Oxford, Health Economics Research Centre Nuffield Department of Population Health<br>Rogers, Chris; University of Bristol, Bristol Trials Centre (CTEU)
**Primary Subject Heading**:	Oncology
Secondary Subject Heading:	Surgery
Keywords:	ONCOLOGY, CHEMOTHERAPY, Thoracic surgery < SURGERY, THORACIC MEDICINE

SCHOLARONE™ Manuscripts

I, the Submitting Author has the right to grant and does grant on behalf of all authors of the Work (as defined in the below author licence), an exclusive licence and/or a non-exclusive licence for contributions from authors who are: i) UK Crown employees; ii) where BMJ has agreed a CC-BY licence shall apply, and/or iii) in accordance with the terms applicable for US Federal Government officers or employees acting as part of their official duties; on a worldwide, perpetual, irrevocable, royalty-free basis to BMJ Publishing Group Ltd ("BMJ") its licensees and where the relevant Journal is co-owned by BMJ to the co-owners of the Journal, to publish the Work in this journal and any other BMJ products and to exploit all rights, as set out in our licence.

The Submitting Author accepts and understands that any supply made under these terms is made by BMJ to the Submitting Author unless you are acting as an employee on behalf of your employer or a postgraduate student of an affiliated institution which is paying any applicable article publishing charge ("APC") for Open Access articles. Where the Submitting Author wishes to make the Work available on an Open Access basis (and intends to pay the relevant APC), the terms of reuse of such Open Access shall be governed by a Creative Commons licence – details of these licences and which Creative Commons licence will apply to this Work are set out in our licence referred to above.

Other than as permitted in any relevant BMJ Author's Self Archiving Policies, I confirm this Work has not been accepted for publication elsewhere, is not being considered for publication elsewhere and does not duplicate material already published. I confirm all authors consent to publication of this Work and authorise the granting of this licence.

For peer review only

Mesothelioma and Radical Surgery 2 (MARS 2): protocol for a multicentre randomised trial comparing (extended) pleurectomy decortication versus no (extended) pleurectomy decortication for patients with malignant pleural mesothelioma

Eric Lim[1]	e.lim@rbht.nhs.uk
Liz Darlison[2]	liz.darlison@uhl-tr.nhs.uk
John G Edwards[3]	john.edwards3@nhs.net
Daisy Elliott[4]	daisy.elliott@bristol.ac.uk
Dean A Fennell[5]	df132@leicester.ac.uk
Sanjay Popat[6]	sanjay.popat@rmh.nhs.uk
Robert Rintoul[7]	robert.rintoul@nhs.net
David Waller[8]	david.waller1@nhs.net
Clinton Ali[9]	clinton.ali@ggc.scot.nhs.uk
Andrea Bille[10]	andrea.bille@gstt.nhs.uk
Liz Fuller[11]	liz.fuller@stft.nhs.uk
Andreea A Ionescu[12]	alina.ionescu2@wales.nhs.uk
Manjusha Keni[13]	manjusha.keni@nhs.net
Alan Kirk[14]	Alan.Kirk@gjnh.scot.nhs.uk
Pek Koh[15]	pek.koh@nhs.net
Kelvin Lau[8]	kelvin.lau@bartshealth.nhs.uk
Talal Mansy[16]	talal.mansy@stees.nhs.uk
Nick A Maskell[17]	nick.maskell@bristol.ac.uk
Richard Milton[18]	richard.milton@leedsth.nhs.uk
Dakshinamoorthy Muthukumar[19]	muthu.kumar@colchesterhospital.nhs.uk
Tony Pope[20]	tony.pope@nhs.net
Amy Roy[21]	amyroy@nhs.net
Riyaz Shah[22]	riyaz.shah@nhs.net
Jonathan Shamash[23]	jonathan.shamash@bartshealth.nhs.uk
Zacharias Tasigiannopoulos[24]	zacharias.tasigiannopoulos@nnuh.nhs.uk
Paul Taylor[25]	paul.taylor@uhsm.nhs.uk
Sarah Treece[26]	sarah.treece@pbh-tr.nhs.uk
Kate Ashton[27]	kate.ashton@bristol.ac.uk
Rosie A Harris[27]	rosie.harris@bristol.ac.uk
Katherine Joyce[27]	katie.joyce@bristol.ac.uk
Barbara Warnes[27]	barbara.warnes@bristol.ac.uk

For peer review only

Nicola Mills[4] nicola.mills@bristol.ac.uk
Elizabeth Stokes[28] elizabeth.stokes@dph.ox.ac.uk
Chris A Rogers on behalf of the MARS 2 Trialists*[27] chris.rogers@bristol.ac.uk

CORRESPONDING AUTHOR:

Professor Eric Lim

Academic Division of Thoracic Surgery

The Royal Brompton Hospital

Sydney Street

London SW3 6NP

United Kingdom

Tel: +44 (0)207 351 8591

Fax: +44 (0)207 351 8560

Email: e.lim@rbht.nhs.uk

1. Academic Division of Thoracic Surgery, The Royal Brompton and Harefield NHS Foundation Trust, London, UK
2. Institute for Lung Health, University Hospitals of Leicester, Leicester, UK
3. Cardiothoracic Centre, Sheffield Teaching Hospitals NHS Foundation Trust, Sheffield, UK
4. Population Health Sciences, University of Bristol, Bristol, UK
5. Cancer Research Centre, University Hospitals of Leicester NHS Trust, Leicester Royal Infirmary, Leicester, UK
6. Department of Medicine – Lung, Institute of Cancer Research, The Royal Marsden NHS Foundation Trust, Fulham Road, London, UK
7. Department of Oncology, University of Cambridge and Papworth Trials Unit Collaboration, Royal Papworth Hospital NHS Foundation Trust, Cambridge, UK
8. Department of Thoracic Surgery, Barts Health NHS Trust, The Royal London Hospital, London, UK
9. The Beatson West of Scotland Cancer Centre, Gartnavel General Hospital, Glasgow, UK
10. Thoracic Surgery, Guy's and St Thomas's NHS Foundation Trust, Guy's Hospital, London, UK
11. Respiratory Medicine, South Tyneside General District Hospital, South Shields, UK
12. Lung Cancer, Aneurin Bevan Health Board (Royal Gwent Hospital), Newport, UK
13. Oncology, Derby Teaching Hospitals NHS Foundation Trust, Derby, UK
14. Department of Thoracic Surgery, Golden Jubilee National Hospital, Glasgow, UK

For peer review only

For peer review only - http://bmjopen.bmj.com/site/about/guidelines.xhtml

15. Department of Oncology, Royal Wolverhampton NHS Trust, Wolverhampton, UK
16. Oncology, South Tees Hospitals NHS Foundation Trust, Middlesbrough, UK
17. Academic Respiratory Unit, University of Bristol, Bristol, UK
18. Thoracic Surgery Department, Leeds Teaching Hospitals NHS Trust, Leeds, UK
19. Oncology, Colchester Hospital University NHS Foundation Trust, Colchester, UK
20. The Clatterbridge Cancer Centre NHS Foundation Trust, Wirral, UK
21. Plymouth Oncology Centre, University Hospitals Plymouth NHS Trust, Plymouth, UK
22. Kent Oncology Centre, Maidstone and Tunbridge Wells NHS Trust, Tunbridge Wells, UK
23. Department of Medical Oncology, Barts Health NHS Trust, The Royal London Hospital, London, UK
24. The Oncology Care Team, Norfolk & Norwich University Hospitals NHS Foundation Trust, Norwich, UK
25. Manchester Lung Cancer Group, Manchester University NHS Foundation Trust, Manchester, UK
26. Heamatology and Oncology Unit, North West Anglia NHS Foundation Trust, Peterborough City Hospital, Peterborough, UK
27. Bristol Trials Centre Clinical Trials and Evaluation Unit, Bristol Medical School, University of Bristol, Bristol, UK
28. Health Economics Research Centre, Nuffield Department of Population Health, University of Oxford, Oxford, UK

WORD COUNT: 3703 (excluding title page, abstract, references, figures and tables)

Number of figures: 1

Number of tables: 1

KEYWORDS: Randomised controlled trial; mesothelioma; surgery; radical surgery; pleurectomy decortication; cancer

For peer review only

ABSTRACT

Introduction: Mesothelioma remains a lethal cancer. To date, systemic therapy with pemetrexed and a platinum drug remains the only licenced standard of care. As the median survival for mesothelioma patients is 12.1 months, surgery is an important consideration to improve survival and/or quality of life. Currently, only two surgical trials have been performed which found that neither extensive (extra-pleural pneumonectomy) or limited (partial pleurectomy) surgery improved survival (although there was some evidence of improved quality of life). Therefore, clinicians are now looking to evaluate pleurectomy decortication, the only radical treatment option left.

Methods and analysis: The MARS 2 study is a UK multicentre open parallel group randomised controlled trial comparing the effectiveness and cost-effectiveness of surgery - (extended) pleurectomy decortication - versus no surgery for the treatment of pleural mesothelioma. The study will test the hypothesis that surgery and chemotherapy is superior to chemotherapy alone with respect to overall survival. Secondary outcomes include health related quality of life, progression free survival, measures of safety (adverse events) and resource use to 2 years. The QuinteT Recruitment Intervention is integrated into the trial to optimise recruitment.

Ethics and dissemination: Research ethics approval was granted by London - Camberwell St. Giles Research Ethics Committee (reference 13/LO/1481) on 7th November 2013. We will submit the results for publication in a peer-reviewed journal.

Trial registration numbers: ISRCTN – ISRCTN44351742 and ClinicalTrials.gov – NCT02040272.

For peer review only

For peer review only - http://bmjopen.bmj.com/site/about/guidelines.xhtml

ARTICLE SUMMARY

Strengths and limitations of this study

- (Extended) pleurectomy decortication is currently offered to patients with mesothelioma on the United Kingdom National Health Service, but it is unknown whether it is a clinically beneficial or cost-effective treatment option. MARS 2 is the first randomised controlled trial to compare this type of surgery with no surgery in this patient population.
- Surgical quality assurance measures will be implemented to ensure that the intervention will be delivered at centres with expertise.
- Patients may come with a pre-conceived perception that surgery will be beneficial, which can lead to crossovers (i.e. patients allocated to no surgery may go on to seek surgery elsewhere). The integrated Quintet Recruitment Intervention supports recruitment staff in responding to patient preferences and conveying balanced information.
- It is not possible to blind participants or the study team, but the primary outcome (survival) is objective.
- Patient pathways vary at different sites. Some flexibility has been worked into the protocol to allow for this.

INTRODUCTION

In the United Kingdom (UK), approximately 2,500 patients are diagnosed each year with pleural mesothelioma,[1] a treatment resistant and lethal cancer of the membranes lining the outer surface of the lung and the inside of the chest wall primarily due to asbestos exposure. Deaths are increasing yearly and are estimated to peak this year.[2] So far, most treatments have proven ineffective. The current standard of care, consisting of 4 to 6 cycles of platinum and pemetrexed chemotherapy, as recommended by the National Institute for Health and Care Excellence (NICE)[3], has been associated with only an additional 3 months of survival.[4] As the median survival for mesothelioma patients is 12.1 months,[4] surgery to remove as much of the disease as possible remains an important consideration to improve survival and/or health related quality of life (HRQoL).[5]

Pleurectomy decortication is the most common surgical procedure for mesothelioma worldwide and is defined as parietal and visceral pleurectomy to remove all gross tumour without diaphragm or pericardial resection.[6] Extended pleurectomy decortication can also be carried out, when parietal and visceral pleurectomy is undertaken to remove all gross

For peer review only - http://bmjopen.bmj.com/site/about/guidelines.xhtml

tumour, including the resection of the diaphragm and/or pericardium. In the document we use the term (extended) pleurectomy decortication to refer to either of the two procedures. The other main types of surgery for mesothelioma are: extra-pleural pneumonectomy, which is defined as en bloc resection of the parietal and visceral pleura with the ipsilateral lung, pericardium, and diaphragm (in cases where the pericardium and/or diaphragm are not involved by tumour, these structures may be left intact) and partial pleurectomy, which is the partial removal of parietal and/or visceral pleura for diagnostic or palliative purposes but leaving gross tumour behind.[6]

So far, no advantage, in terms of survival, has been observed with any type of surgery in randomised controlled trials (RCTs) conducted to date. The MARS feasibility study (ISRCTN95583524), a trial of extra-pleural pneumonectomy with adjuvant haemothorax irradiation, concluded that surgery was unlikely to offer either an improvement to survival or HRQoL and possibly harmed patients.[7] MesoVATS (ISRCTN34321019) concluded that partial pleurectomy did not improve survival, although it showed that patients in the better prognostic group, had improved HRQoL after 6 months.[8]

Suitable patients, both in the UK and internationally, are currently offered pleurectomy decortication as it is considered to carry less morbidity compared to the more extensive extra-pleural pneumonectomy but still achieves complete macroscopic resection which partial pleurectomy does not.[9-11] However, we do not know if (extended) pleurectomy decortication in conjunction with chemotherapy will improve survival compared to the current standard of care (chemotherapy alone). In the absence of RCTs, (extended) pleurectomy decortication may continue to be offered despite a lack of high-quality evidence of clinical efficacy or any evidence on cost-effectiveness.

Aims and objectives

MARS 2 is a UK wide multicentre RCT which will test the hypothesis that (extended) pleurectomy decortication and chemotherapy is superior to chemotherapy alone with respect to overall survival for patients with pleural mesothelioma.

Specific objectives are to estimate:

A. The difference between groups in overall survival.
B. The difference between groups with respect to a range of secondary outcomes including HRQoL, progression free survival and measures of safety (adverse health events).

For peer review only - http://bmjopen.bmj.com/site/about/guidelines.xhtml

C. The cost effectiveness of (extended) pleurectomy decortication compared to no surgery.

METHODS AND ANALYSIS

Trial design

MARS 2 is a multicentre, non-blinded parallel two-group, pragmatic RCT of surgery and chemotherapy versus chemotherapy alone for suitable patients with mesothelioma.

An internal pilot funded by Cancer Research UK (award ref: C27967/A15895) and coordinated by the Papworth Trials Unit Collaboration, demonstrated the feasibility of recruitment across 14 medical sites and 2 joint medical and surgical sites of excellence, as the target of 50 participants recruited within a 24 month period was achieved.

Since the end of the pilot phase in December 2016 an additional 8 medical, 1 surgical and 2 joint medical and surgical sites have been opened for the full trial. In addition, the full trial will provide recruiting sites with the support of an integrated QuinteT Recruitment Intervention (QRI)[12-14] to optimise recruitment and retention.

Setting, centre and surgeon eligibility

This study is taking place in National Health Service (NHS) secondary care centres, including teaching and district general hospitals.

To be eligible as a medical site, the centre must:

i) be an NHS Trust with access to a multidisciplinary team (MDT) to discuss patients with mesothelioma;
ii) have a track record of treating patients with mesothelioma

To be eligible as a surgical site, the centre must:

i) be an NHS Trust with an established mesothelioma MDT;
ii) have a minimum of 2 named mesothelioma surgeons participating in the trial.

All surgeons participating in the full trial must be accredited by; i) self-reporting a minimum of 5 cases in which they have performed (extended) pleurectomy decortication; ii) observing the procedure being undertaken at an established MARS 2 surgical site; iii) having a surgeon from the pilot phase observe their first MARS 2 procedure undertaken; and iv)

For peer review only - http://bmjopen.bmj.com/site/about/guidelines.xhtml

having one randomly selected MARS 2 operation between procedures 5 and 10 observed by a surgeon from the pilot phase to ensure fidelity.

Patients from all medical (only) sites are referred to a trial-accredited surgical site for computed tomography (CT) assessment of eligibility, further discussion about the study, and surgery (if randomised to this group).

Trial population

The target population are patients with a diagnosis of epithelioid, sarcomatoid or biphasic mesothelioma. Patients will be eligible to take part if ALL of the following apply:

- Adult aged ≥ 16 years of age;
- Tissue (cytology or histology) confirmed epithelioid, sarcomatoid or biphasic mesothelioma, as reviewed by MDT to be of sufficient certainty to recommend chemotherapy as treatment;
- Disease confined to one hemi-thorax based on CT assessment;
- Disease deemed surgically resectable by a surgeon at a MARS 2 surgical site;
- Deemed fit for surgery by a surgeon at a MARS 2 surgical site;
- Capacity to provide written informed consent to participate in the trial.

Patients will not be eligible if they have:

- Severe shortness of breath (Eastern Cooperative Oncology Group (ECOG) status ≥ 2; or pre-operative forced expiratory volume after one second (FEV1) or transfer factor of the lung for carbon monoxide (TLco) less than 20%);
- Severe heart failure (NYHA III or IV, or ejection fraction less than 30% by echocardiogram);
- End stage kidney failure requiring dialysis;
- Liver failure (e.g. encephalopathy and/or coagulation abnormalities);
- Any other serious concomitant disorder that would compromise participant safety during surgery;
- Prisoner;
- Patient lacks capacity to consent;
- Existing co-enrolment in another interventional study that aims to improve survival.

Patient approach, consent and randomisation

For peer review only - http://bmjopen.bmj.com/site/about/guidelines.xhtml

The local research team at the medical site will take written informed consent from participants. In addition to the main study, the team may also seek consent for audio-recording of consultations and participation in interviews, for QRI purposes. Participants will then receive two cycles of chemotherapy (standard care) and have a further CT scan to confirm eligibility (i.e. disease still resectable) before being randomised, using a secure web-based randomisation system (Sealed Envelope https://sealedenvelope.com).

Participants will be randomised in a 1:1 ratio. Minimisation (with a random component) will be applied for selected baseline variables (age, performance status and cell type) that influence survival, in addition to stratification by recruiting site to ensure that the cohorts are as balanced as possible.

For peer review only

Trial interventions

Patients will be randomised to receive one of the following interventions:

- *(Extended) pleurectomy decortication and chemotherapy;* two cycles of platinum and pemetrexed chemotherapy followed by surgery and then up to four cycles of the same chemotherapy.
- *Chemotherapy alone (control intervention);* Up to six cycles of platinum and pemetrexed chemotherapy alone (current standard of care).

The trial schema is illustrated in figure 1.

After randomisation, any changes in the choice of chemotherapy, addition of other agents or entry into therapeutic trials (e.g. immunotherapies) will be permitted for patients with progressive disease. At the time of trial design, there was no national consensus on post-operative prophylactic radiotherapy so it was decided that irradiation to thoracic procedure sites may be undertaken for MARS 2 patients. Patients in both groups can also receive further surgery, including thoracic, if it is without radical intent. The aim is to conduct a pragmatic trial whilst closely monitoring uptake of additional therapies, studies or surgeries in order to account for them in the trial analyses, if required.

Primary and secondary outcomes

The primary outcome is survival, calculated from randomisation date (randomisation occurs after the first two cycles of chemotherapy). All participants will be followed up to the end of the trial (minimum of two years after randomisation).

For peer review only - http://bmjopen.bmj.com/site/about/guidelines.xhtml

Secondary outcomes have been selected to assess the efficacy of the two approaches. Secondary outcomes are 1) progression free survival to the end of the trial (minimum of two years after randomisation); 2) serious adverse health events to two years after randomisation; 3) disease specific and generic HRQoL using the following validated questionnaires: European Organisation for Research and Treatment of Cancer Quality of Life Questionnaire (EORTC QLQ-C30) – to assess the HRQoL of cancer patients; and EuroQol EQ-5D-5L[15 16] – a widely used generic measure of HRQoL (both of these will be measured at baseline, pre-randomisation, and 6 weeks, 6, 12, 18 and 24 months post-randomisation) and 4) healthcare resource use to the end of the study: chemotherapy cycles and initial surgical admission (for chemotherapy plus surgery group), and further resources measured at 6 weeks post-randomisation then every 6 months, with a final follow up at the end of the study if not followed up in the previous 4 months.

Data collection

The schedule for data collection for the study is shown in table 1. Data will be collected onto purpose-designed case report forms (CRFs) and participant completed questionnaires and entered onto a bespoke database for data cleaning and analysis. Access to the database will be via a secure password-protected web-interface hosted on an NHS server. Data about adverse events will be collected and reported in accordance with Sponsor's and regulatory requirements.

For peer review only

For peer review only - http://bmjopen.bmj.com/site/about/guidelines.xhtml

Table 1 Data collection

	Pre-randomisation				Post-randomisation							
	Screening	Baseline	2 cycles chemo-therapy	End of chemo-therapy cycle 2	Surgical admission *	Up to 4 cycles chemo-therapy	Follow-up					
							6 w	6 m	12 m	18 m	24 m	Every 6 m and end of trial**
Screening log	ü			ü								
CT scan	ü***			ü								
Informed consent		ü										
Demography, medical history, blood test results		ü										
Lung function tests	ü	ü****										
HRQoL		ü		ü			ü	ü	ü	ü	ü	
Chemotherapy treatment given***			ü			ü						
Surgery and in hospital post-operative data*****					ü							
Adverse events					ü		ü	ü	ü	ü	ü	
Patient reported resource and health service use							ü	ü	ü	ü	ü	ü

* Patients allocated to surgery and/or receiving surgery only

For peer review only

** If not within previous 4 months
*** Previous CT scan to be used (not to be done again specifically for the trial protocol)
**** Only one assessment of lung function is needed so if this has been done prior to screening there is no need for another test at baseline
***** Including resource and health service use

Risk of bias

Participants and clinical personnel cannot be blinded to allocation due to the nature of the study intervention. However, standard local protocols will be followed in terms of patient care. The patient information leaflet and conversations with MARS 2 site staff will describe and balance the potential benefits and risks of both having and not having surgery. Therefore, this approach will reduce participant's expectations that one or other treatment protocol will lead to a more favourable result.

In addition, the study's primary outcome is an objective measure (survival), and clear definitions of each secondary outcome measure will be provided to trial personnel. The HRQoL follow-up questionnaires may be more at risk of bias than other measures, but patients will not have had this surgery previously and as such, should not have any expectation regarding its effect on their HRQoL. Missing outcome data will be minimised, as survival and progression free survival data can be obtained from hospital records. Losses to follow up will be minimised by maintaining regular contact with participants (by telephone and post) to complete follow-up questionnaires. Non-adherence to randomised allocation will be documented. Bias in the reported results will be minimised by having pre-specified outcomes in the trial protocol and a pre-specified analysis plan.

Sample size

The total sample size has been set at 328 participants (164 per group). The patients randomised in the pilot trial will contribute to the total sample size. The study will have 80% power to detect a hazard ratio (HR) of 0.7 at 5% statistical significance (2-sided), modelled on a published assumption of a median survival time of 16.8 months in mesothelioma patients who were fit enough to receive surgery, but did not have it[17] and allowing for 10% cross-over from the medical to surgery groups (as noted in previous trials such as MARS 1[7]). Cross-over will be minimised through instruction (i.e. recruit only patients who have equipoise from the outset) and education.

For peer review only - http://bmjopen.bmj.com/site/about/guidelines.xhtml

The relative difference of 30% (HR 0.7) was regarded as the minimally important difference for patients and clinicians to choose surgery given the risks of the procedure. The figure was chosen by the trial's patient and public involvement (PPI) group. The possibility that survival could be worse with surgery was also discussed, and a relative difference of 30% also regarded as an appropriate difference to indicate harm, therefore a two-tailed test for superiority was agreed.

Patient and Public Involvement

Patient and public representatives were involved from inception and advised on the trial design of MARS 2, the identification of the choice of the primary outcome and defined the minimally important difference in relative survival.

The study team have continuing engagement with the Royal Brompton Hospital Cancer Consortia PPI group that consists of patients and carers who have undergone surgery for lung cancer and mesothelioma to advise on patient orientated questions that arise from the trial conduct. One patient from the PPI group, a mesothelioma survivor, has agreed to sit on the Trial Steering Committee. The PPI group will also be involved in the dissemination of study results.

Integrated QuinteT Recruitment Intervention (QRI)

Recruitment to RCTs can be challenging[18], particularly for surgical trials.[19] An integrated QRI will therefore be employed during the main study phase to optimise recruitment and retention. The aim of the QRI is to understand the recruitment process and how it operates in clinical centres, so that sources of recruitment difficulties can be identified, and suggestions made to change aspects of design, conduct, organisation or training.

A multi-faceted, flexible approach will be used to investigate site-specific or wider recruitment obstacles. These will comprise the following:

- Mapping of eligibility and recruitment pathways to collate basic data about the levels of eligibility and recruitment, and identify points at which patients opt in or out of the trial;
- In-depth, semi-structured interviews with a purposive sample of staff members involved with aspects of trial design/management and recruitment across centres, and patients eligible for recruitment to the trial. Interviews will explore participants' perspectives of the trial, views on the presentation of study information,

For peer review only - http://bmjopen.bmj.com/site/about/guidelines.xhtml

understanding of trial processes (e.g. randomisation), and reasons underlying decisions to accept or decline the trial. In addition, interviews with staff and other individuals involved in the trial will explore perspectives on the trial design and protocol; views about the evidence on which the trial is based; perceptions of uncertainty/equipoise for themselves and their colleagues; methods for identifying eligible patients; views on eligibility, and examples of actual recruitment successes and difficulties. Interview topic guides will be used to ensure similar topic areas are covered across interviews, while still providing the scope for participants to raise issues of pertinence to them.

- Audio recording of consultations between healthcare staff and potentially eligible patients across centres to understand the recruitment process at each centre and to identify and investigate the challenges to recruitment. The QRI researcher will listen to and qualitatively analyse the appointments, documenting instances such as unclear, insufficient or imbalanced information provision and unintentional transferring of clinician treatment preferences to patients.
- Observation of Trial Management Group (TMG) and investigator meetings to gain an overview of trial conduct and overarching challenges (logistical issues, etc.).

An account of the anonymised findings from all the data will be fed back to the Chief Investigator and TMG. The data will be used by the QRI team to provide supportive and confidential individual and group feedback to recruiters to help them to communicate equipoise, balance treatment options and explain to patients the benefits and purposes of trial participation, whilst optimising informed consent.

Statistical analyses

The data will be analysed for randomised patients according to intention to treat and follow Consolidated Standards of Reporting Trials (CONSORT) guidelines. Analyses will be adjusted for site and for design factors included in the cohort minimisation (e.g. age, performance status and cell type).

Survival time and progression free survival time from randomisation will be compared using survival methods, allowing for censoring of any participant who is either alive or lost to follow-up at the end of the follow-up period. Patient reported outcome scores (HRQoL EQ-5D-5L and QLQ-C30) will be compared using a mixed regression model, adjusted for baseline measures where appropriate. Changes in treatment effect with time will be assessed by adding a treatment x time interaction to the model and comparing models using a likelihood ratio test. Deaths will be accounted for by modelling survival and HRQoL jointly.

For peer review only - http://bmjopen.bmj.com/site/about/guidelines.xhtml

Model fit will be assessed using standard methods and alternative models and/or transformations will be explored if appropriate. Treatment differences and 95% confidence intervals will be reported.

Missing data on patient questionnaires will be dealt with according to the scoring manuals. Multiple imputation methods will be used if greater than 5% of cases have missing data, otherwise complete case analysis will be undertaken. Compliance rates will be reported, including the number of participants who have withdrawn from the study, have been lost to follow-up or died. Causes of death for trial participants will be recorded.

Frequencies of adverse events will be described. The proportion of participants experiencing one or more serious adverse events in the two-year follow-up period will be compared using a generalised linear model.

Two subgroup analyses are planned: i) comparing primary and secondary outcomes by the experience level of the surgical site; and ii) comparing the primary outcome by type of mesothelioma (epithelioid, sarcomatoid or biphasic). An exploratory analysis investigating the effect of surgeon (surgical group only) will be performed for the primary outcome.

No interim analyses are planned. The primary analysis will take place when follow-up is complete for all recruited participants.

Economic evaluation

The economic evaluation will compare the costs and effects of surgery versus no surgery, following established guidelines as set out by NICE.[20] The within-trial cost-effectiveness analysis will be undertaken from an NHS and personal social services perspective, with a time horizon from time of consent to 24 months post-randomisation. The primary outcome measure for the economic evaluation will be quality adjusted life years (QALYs), estimated using the EuroQol EQ-5D-5L at each follow up timepoint.[15 16] Resource use data collection will be integrated into the trial CRFs for chemotherapy cycles and surgery (if applicable, this will include details of the surgical procedure, length of stay in hospital by level of care, and post-operative complications) and be collected at each follow up timepoint.

Unit costs will be sought to value resource use data, and the total costs per participant calculated. Responses to the EQ-5D-5L will be assigned valuations according to NICE guidance at the time of analysis,[21] and combined with survival to calculate QALYs gained per participant. Missing resource use and EQ-5D-5L data will be handled using multiple

For peer review only - http://bmjopen.bmj.com/site/about/guidelines.xhtml

imputation methods.[22] From the average costs and QALYs gained in each trial group, the incremental cost-effectiveness ratio will be derived, producing an incremental cost per QALY gained of surgery compared to no surgery. Sensitivity analyses will assess the impact of varying key parameters on baseline cost-effectiveness results. Results will be expressed in terms of a cost-effectiveness acceptability curve, which indicates the likelihood that surgery is cost-effective for different levels of willingness to pay for health gain.

Ethics and dissemination

The study intervention is already routinely used in the NHS. This study has been reviewed and given favourable opinion by the London - Camberwell St. Giles Research Ethics Committee (REC; reference 13/LO/1481) on 7th November 2013. The pilot study was managed by Papworth Trials Unit Collaboration and the main trial is managed by the Bristol Trials Centre Clinical Trials and Evaluation Unit and sponsored by Royal Brompton & Harefield NHS Foundation Trust. Each participant has the right to withdraw at any time. In addition, the investigator may withdraw the participant from their allocated treatment group if a clinical reason for not performing the surgical intervention is discovered. If a participant wishes to withdraw, any data already collected will be included in the study analyses, unless the participant expresses a wish for their data to be excluded. Withdrawing patients will be asked if they would continue in follow up and complete the requisite questionnaires. Participants who choose to withdraw from the study will be treated according to their hospitals' standard procedures.

The findings will be disseminated by usual academic channels, i.e. presentation at international meetings and peer-reviewed publications. A full report for the funder will be written on completion of the study and a lay summary of the results provided to patients.

Major changes to protocol

Since the first study protocol was approved by the REC (the current version is v6.0, 10 April 2019), the following changes have been made:

- Qualitative assessment sub-study added, as part of the pilot phase only.
- The EuroQol EQ-5D-5L was added.
- Updates to transition from pilot phase to main study, including addition of the integrated QRI and economic evaluation, and removal of the collection of blood and tissue samples, and one of the disease specific questionnaires – the EORTC QLQ LC-13.

For peer review only - http://bmjopen.bmj.com/site/about/guidelines.xhtml

- Length of follow up extended from two years until the end of the study for all participants to ensure that the study has 80% power.
- Video-recording aspect of the surgical quality assurance removed as this was deemed impractical by sites, and it was agreed that it was unnecessary by the Data Safety and Monitoring Committee and the Trial Steering Committee, acknowledging the other surgical quality assurance measures that are in place.

Study progress

Recruitment started in May 2015 and 304 patients have been randomised so far (correct on 25 March 2020). 66 patients from the pilot study are included in this figure. Recruitment will continue until 1st June 2020.

The full protocol is available from:
https://www.journalslibrary.nihr.ac.uk/programmes/hta/1518831/

ADDITIONAL FIGURES

Figure 1. Trial schema showing the recruitment pathway for the MARS 2 study

ABBREVIATIONS

CONSORT- Consolidated Standards of Reporting Trials
CRF – Case Report Form
CT- computed tomography
ECOG- Eastern Cooperative Oncology Group
EORTC- European Organisation for Research and Treatment of Cancer
FEV1- forced expiratory volume after one second
HR- hazard ratio
HRQoL- health related quality of life
ISRCTN - International Standard Randomised Controlled Trials Number
MDT – multidisciplinary team
NHS- National Health Service
NICE- National Institute for Health and Care Excellence
NIHR- National Institute for Health Research
NYHA – New York Heart Association
PPI- patient and public involvement
QALY- quality adjusted life years
QLQ- quality of life questionnaire
QuinteT – Qualitative research integrated within trials
QRI- QuinteT Recruitment Intervention
RCT- randomised controlled trial
REC- Research Ethics Committee
TLco- transfer factor of the lung for carbon monoxide
TMG – Trial Management Group

For peer review only - http://bmjopen.bmj.com/site/about/guidelines.xhtml

UK- United Kingdom

Acknowledgements

The MARS 2 trial is sponsored by The Royal Brompton and Harefield NHS Foundation Trust. The sponsor will be responsible for the oversight of the MARS 2 study and to ensure the trial is managed appropriately. We want to thank the large teams at each hospital who work on the MARS 2 study (representatives from each are listed below). We would also like to thank Dr Fiona McDonald from the Royal Marsden Hospital, Dr Nagmi Qureshi from Papworth Hospital and Professor Simon Padley from the Royal Brompton Hospital for their radiotherapy advisory roles. Thank you also to Professor Andrew Nicholson for his histopathology advisory role for MARS 2.

Collaborators

***MARS 2 Trialists**

Project management team members:

Professor Eric Lim, Chief Investigator
Professor Chris Rogers, Methodological Lead and Statistician
Kate Ashton, Clinical Trial Coordinator
Athanasia Gravani, Clinical Trial Coordinator
Holly McKeon, Clinical Trial Coordinator
Wendy Underwood, Clinical Trial Coordinator
Dr Barbara Warnes, Clinical Trial Coordinator
Katherine Joyce, Assistant Clinical Trial Coordinator
Rachel Brophy, Assistant Clinical Trial Coordinator
Rosie Harris, Medical Statistician
Dr Nicola Mills, Senior Research Fellow, Quintet Research Intervention
Dr Daisy Elliott, Research Fellow, Quintet Research Intervention
Nicola Farrar, PhD Student, Quintet Research Intervention
Dr Elizabeth Stokes, Health Economist

Papworth Trials Unit Collaboration (pilot study):

Robert Rintoul, Director of Papworth Trials Unit Collaboration and Honorary Consultant Respiratory Physician
Victoria Hughes, Senior R&D Manager
Jane Elliott, Clinical Project Manager
Claire Matthews, Trial Manager
Philip Noyes, Trial Administrator

Participating Sites Members: pilot study and main trial

University Hospitals of Leicester NHS Trust – *medical and surgical site (opened 22/04/2015)*

Dean Fennell, Principal Investigator, Chair of Thoracic Medical Oncology
Apostolos Nakas, Consultant Thoracic Surgeon
Louise Nelson, Research Nurse

Sheffield Teaching Hospitals NHS Foundation Trust *– medical and surgical site (opened 13/05/2015)*

John Edwards, Principal Investigator, Consultant Thoracic Surgeon
Sara Tenconi, Consultant Thoracic Surgeon

For peer review only

Laura Socci, Consultant Thoracic Surgeon
Hilary Wood, Data Manager
Helena Hanratty, Research Nurse
Helena Stanley, Mesothelioma UK Clinical Nurse Specialist
South Tyneside and Sunderland NHS Foundation Trust *– medical site (opened 19/06/2015)*
Liz Fuller, Principal Investigator, Consultant Respiratory Physician
Judith Moore, Clinical Trials Officer
Papworth Hospital NHS Foundation Trust *– medical site (opened 01/07/2015)*
Robert Rintoul, Principal Investigator, Honorary Consultant Respiratory Physician
Suzanne Miller, Clinical Trials Coordinator
Amy Gladwell, Clinical Trials Administrator
Jenny Castedo, Research Nurse
Amanda Stone, Senior Research Nurse
Colchester Hospital University NHS Foundation Trust *– medical site (opened 03/11/2015)*
Dakshinamoorthy Muthukumar, Principal Investigator, Consultant Oncologist
Charlotte Ingle, Co-Investigator, Consultant Clinical Oncologist
Hayley Hewer, Research Nurse
South Tees Hospitals NHS Foundation Trust *– medical site (opened 16/11/2015)*
Talal Mansy, Principal Investigator, Consultant Medical Oncologist
Louise Li, Co-Investigator, Consultant Medical Oncologist
Eleanor Aynsley, Co-Investigator, Consultant Clinical Oncologist
Andrea Watson, Clinical Trials Coordinator
Charlotte Jacobs, Clinical Trials Coordinator
The Clatterbridge Cancer Centre NHS Foundation Trust *– medical site (opened 25/11/2015)*
Tony Pope, Principal Investigator, Consultant in Clinical Oncology
Alison Hassall, Research Nurse
Masuma Begum, Clinical Trials Assistant
University Hospitals of Derby and Burton NHS Foundation Trust *– medical site, (opened 26/11/2015)*
Manjusha Keni, Principal Investigator, Consultant Clinical Oncologist
Christopher Worth, Cancer Clinical Research Nurse Lead
Ellie Piggott, Research Practitioner
Elizabeth Nadin, Research Practitioner
Leeds Teaching Hospitals NHS Trust – *medical site (opened 02/12/2015)*
Richard Milton, Principal Investigator, Consultant Thoracic Surgeon
Victoria Ashford-Turner, Research Nurse
Matthew Callister, Respiratory Consultant
Manchester University NHS Foundation Trust *– medical site (opened 21/12/2015)*
Paul Taylor, Principal Investigator
Yvonne Summers, Sub Investigator
Raffaele Califano, Sub Investigator
Laura Cove-Smith, Sub Investigator
Matt Evison, Sub Investigator
Maria Blinston, Research Nurse
Sara Waplington, Clinical Trials Coordinator

For peer review only - http://bmjopen.bmj.com/site/about/guidelines.xhtml
For peer review only

Amal Ismail, Clinical Trials Administrator
Rachel Chant, Research Nurse
Asmita Desai, Research Nurse
Juliette Novasio, Clinical Trials Manager
Marie Kirwan, Research Nurse
The Royal Wolverhampton NHS Trust *– medical site (opened 04/01/2016)*
Pek Koh, Principal Investigator, Consultant Clinical Oncologist
Ian Morgan, previous Principal Investigator, Consultant Cardiothoracic Surgeon
Victoria Lake, Research Nurse
Nichola Harris, Research Nurse
Royal Gwent Hospital, Aneurin Bevan University Health Board *– medical site (opened 08/02/2016)*
Andreea Alina Ionescu, Principal Investigator, Consultant Respiratory Physician
Simon Hodge, Research Nurse
The Royal Marsden NHS Foundation Trust *– medical site (opened 08/04/2016)*
Prof Sanjay Popat, Principal Investigator, Consultant Medical Oncologist
Chelsea sub-site
Dr Nadia Yousaf, Sub-Investigator
Dr Nadza Tokaca, Sub-Investigator
Dr Adam Januszewski, Sub-Investigator
Dr Avani Athauda, Sub-Investigator
Dr Anisha Ramessur, Sub-Investigator
Dr Emily Grist, Sub-Investigator
Dr Niamh Colman, Sub-Investigator
Dr Michael Flynn, Sub-Investigator
Joan Joyce, Research Nurse
Sarah Vaughan, Research Nurse
Maria Piga, Senior Research Nurse
Derya Sahin, Senior Research Nurse
Agnieszka Yongue, Senior Trials Coordinator
Emma Turay, Clinical Trials Administrator
Sutton sub-site
Prof Mary O'Brien, Sub-Investigator
Dr Jaishree Bhosle, Sub-Investigator
Dr Rajiv Kumar, Sub-Investigator
Dr Charlotte Milner-Watts, Sub-Investigator
Dr Jessica Brown, Sub-Investigator
Dr David Walder, Sub-Investigator
Dr Alexandros Georgiou, Sub-Investigator
Dr Xiaorong Wu, Sub-Investigator
Dr Naila Kaudeer, Sub-Investigator
Dr Kroopa Joshi, Sub-Investigator
Dr Michael Davidson, Sub-Investigator
Dr Shelize Khakoo, Sub-Investigator
Bee Ayite, Research Nurse
Kathryn Priest, Research Nurse
James Dobbyn, Senior Research Nurse
Vasanthi Prathapan, Senior Research Nurse

For peer review only

For peer review only - http://bmjopen.bmj.com/site/about/guidelines.xhtml

Deborah McCrimmon, Research Radiographer
Natalie Ash, Assistant Practitioner
Alison Norton, Senior Clinical Trial Coordinator
Bianca Peet, Senior Trials Manager
Libby Hennessy, Clinical Trials Administrator
Rosemary Johnson, Clinical Trials Administrator
Laura White, Clinical Trials Administrator
Kingston sub-site
Dr Edward Armstrong, Sub-Investigator
Dr Maria Coakley, Sub-Investigator
Dr Scott Shepherd, Sub-Investigator
Dr Narda Chaabouni, Sub-Investigator
Dr Katherina Sreter, Sub-Investigator
Dr Vasileios Angelis, Sub-Investigator
Dr Mariko Morishita, Sub-Investigator
Dr Jose Roca, Sub-Investigator
Mary Jane de los Reyes Lauigan, Research Nurse
Katrin Sainudeen, Clinical Trials Practitioner
Helen Morgan, Clinical Trials Administrator
Peterborough City Hospital, North West Anglia NHS Foundation Trust *– medical site (opened 16/05/2016)*
Dr Sarah Treece, Principal Investigator, Consultant Clinical Oncologist
Dr Abigail Hollingdale, Co-Investigator, Consultant Clinical Oncologist
Chloe Eddings, Study Co-ordinator/Research Nurse
Holly Warman, Study Co-ordinator/Clinical Trials Assistant

Participating Sites Members: main trial only
Barts Health NHS Trust *– medical and surgical site (opened 05/06/2017)*
Kelvin Lau, Principal Investigator, Consultant Thoracic Surgeon
David Waller, Consultant Thoracic Surgeon
Jeremy Steele, Consultant Medical Oncologist
Jo Hargrave, Mesothelioma Clinical Nurse Specialist
Resmi Jayachandran, Senior Clinical Trials Practitioner
Pratistha Panday, Clinical Trials Assistant
The Beatson West of Scotland Cancer Centre, Greater Glasgow Health Board *– medical site (opened 14/07/2017)*
Clinton Ali, Principal Investigator, Consultant Medical Oncologist
Austin McInnes, Clinical Trial Coordinator
Golden Jubilee National Hospital *– surgical site (opened 14/07/2017)*
Alan Kirk, Principal Investigator, Consultant Thoracic Surgeon
Rocco Bilancia, Consultant Thoracic Surgeon
Julie Buckley, Research Nurse
Elizabeth Boyd, Research Nurse
North Bristol NHS Trust *– medical site (opened 28/02/2018)*
Nick Maskell, Principal Investigator, Consultant in Respiratory Medicine
Natalie Zahan-Evans, Research Nurse
Anna Morley, Senior Research Nurse

For peer review only - http://bmjopen.bmj.com/site/about/guidelines.xhtml

***Norfolk and Norwich University Hospitals NHS Foundation Trust** – medical site (opened 12/06/2018)*
Zacharias Tasigiannopoulos, Principal Investigator, Consultant Clinical Oncologist
Adela Dann, Clinical Trials Practitioner
Eleanor Mishra, Consultant Respiratory Physician
Pinelopi Gkogkou, Consultant Clinical Oncologist
***University Hospitals Plymouth NHS Trust** – medical site (opened 16/07/2018)*
Amy Roy, Principal Investigator, Consultant Clinical Oncologist
Irene Harvey, Research Nurse
Hilary Congdon, Research Nurse
***Barking, Havering and Redbridge University Hospitals NHS Trust** – medical site (opened 24/07/2018)*
Jonathan Shamash, Principal Investigator, Consultant Medical Oncologist
Alison Ray, Clinical Trials Nurse
***Guy's and St. Thomas' NHS Foundation Trust** – medical and surgical site (opened 10/08/2018)*
Andrea Bille, Principal Investigator, Consultant Thoracic Surgeon
Jehan Mansi, Clinical Trials Practitioner
Amy Quinn, Clinical Research Nurse
***Oxford University Hospitals NHS Foundation Trust** – medical site (opened 19/10/2018)*
Najib M Rahman, Principal Investigator, Consultant and Senior Lecturer in Respiratory Medicine
Jack Seymour, Research Nurse
Hannah Ball, Mesothelioma Nurse
Meenali Chitnis, Consultant Clinical Oncologist
***Maidstone and Tunbridge Wells NHS Trust** – medical site (opened 19/10/2018)*
Riyaz Shah, Principal Investigator, Consultant Medical Oncologist
Eirini Petroyannou, Research Nurse
Kimberley Snoad, Clinical Trials Administrator
Monica Tavares Barbosa, Research Nurse
***University Hospitals Birmingham NHS Foundation Trust** – medical site (opened 03/01/2019)*
Professor Gary Middleton, Principal Investigator, Professor in Medical Oncology
Philip Earwaker, Consultant Medical Oncologist
Haider Abbas, Consultant Medical Oncologist
Parminder Sohal, Oncology Research Sister

Independent Trial Steering Committee members
Prof Marcus Flather MBBS FRCP (Chair), Clinical Professor in Medicine; Dr Paul Beckett, Consultant Respiratory Physician; Miss Carol Tan, Consultant Thoracic Surgeon - has declared the following competing interest: ethicon endostaplers – consultancies; Prof Fergus Gleeson FRCP FRCR, Professor of Radiology and Consultant Radiologist; Prof Fergus MacBeth, Consultant Oncologist; Hon Dr Mavis Nye, Patient Advocate; Dr Harvey Pass, Professor of Thoracic Oncology; Dr Pauline Leonard, Consultant Medical Oncologist – has declared the following competing interests: Teva, Amgen, LCO Pharma; Prof Tom Treasure MS MD FRCS FRCP, Clinical Operations Research Unit.
Unless otherwise stated above, committee members have declared no competing interests.

For peer review only - http://bmjopen.bmj.com/site/about/guidelines.xhtml

Independent Data Monitoring and Safety Committee members

Prof Linda Sharples (Chair), Professor of Medical Statistics; Prof Valerie Rusch, Consultant Thoracic Surgeon; Prof Mark Britton, Consultant Physician; Dr Robin Rudd, London Lung Cancer Group; Prof Joseph S. Friedberg MD FACS, Professor of Surgery and Surgeon-in-Chief; Prof Peter Goldstraw, Emeritus Professor of Thoracic Surgery.
Unless otherwise stated above, committee members have declared no competing interests.

Contributors

EL - Study design, preparation and drafting of protocol and manuscript, Chief Investigator for the trial; LD - Study design, preparation of protocol and review of manuscript; JGE - Study design, preparation of protocol and review of manuscript, Principal Investigator and acquired data for the study; DE - Design of integrated qualitative study, preparation of study protocol, review of manuscript; DAF - Study design, preparation of protocol and review of manuscript, Principal Investigator and acquired data for the study; SP - Study design, preparation of protocol and review of manuscript, Principal Investigator and acquired data for the study; RR - Study design, preparation of protocol and review of manuscript, Principal Investigator and acquired data for the study; DW - Study design, preparation of protocol and review of manuscript, acquired data for the study; CA - Review of manuscript, Principal Investigator and acquired data for the study; AB - Review of manuscript, Principal Investigator and acquired data for the study; LF - Review of manuscript, Principal Investigator and acquired data for the study; AAI - Review of manuscript, Principal Investigator and acquired data for the study; MK - Review of manuscript, Principal Investigator and acquired data for the study; AK - Review of manuscript, Principal Investigator and acquired data for the study; PK - Review of manuscript, Principal Investigator and acquired data for the study; KL - Review of manuscript, Principal Investigator and acquired data for the study; TM - Review of manuscript, Principal Investigator and acquired data for the study; NAM - Review of manuscript, Principal Investigator and acquired data for the study; RM - Review of manuscript, Principal Investigator and acquired data for the study; DM - Review of manuscript, Principal Investigator and acquired data for the study; TP- Review of manuscript, Principal Investigator and acquired data for the study; AR - Review of manuscript, Principal Investigator and acquired data for the study; RS - Review of manuscript, Principal Investigator and acquired data for the study; JS - Review of manuscript, Principal Investigator and acquired data for the study; ZT - Review of manuscript, Principal Investigator and acquired data for the study; PT - Review of manuscript, Principal Investigator and acquired data for the study; ST - Review of manuscript, Principal Investigator and acquired data for the study; KA - Study design, preparation and drafting of protocol and manuscript, oversaw study conduct and acquisition of data; RAH - Statistical analysis plan, review of manuscript; KJ - Preparation and drafting of manuscript, oversaw study conduct and acquisition of data; BW- Drafting of manuscript, oversaw study conduct and acquisition of data; NM - Conduct of integrated qualitative study, preparation of study protocol, review of manuscript; ES - Design of health economic component, preparation of study protocol, review of manuscript; CAR - Study design, sample size and statistical analysis plan, drafting of protocol, review of manuscript.

Funding

This research is funded by the National Institute for Health Research (NIHR) Health Technology Assessment (HTA) Programme (project number 15/188/31). The pilot study was

For peer review only - http://bmjopen.bmj.com/site/about/guidelines.xhtml

funded by Cancer Research UK and Mesothelioma UK has contributed towards patient travel expenses.

This study was designed and delivered in collaboration with the Bristol Trials Centre Clinical Trials and Evaluation Unit (CTEU), a UK Clinical Research Collaboration (UKCRC) registered clinical trials unit which, as part of the Bristol Trials Centre (BTC), is in receipt of NIHR Clinical Trials Unit (CTU) support funding.

Daisy Elliott was supported by the NIHR Biomedical Research Centre at University Hospitals Bristol NHS Foundation Trust and the University of Bristol. The views expressed in this publication are those of the author(s) and not necessarily those of the NHS, the National Institute for Health Research or the Department of Health and Social Care.
Sanjay Popat acknowledges NHS funding to the Royal Marsden Hospital / Institute of Cancer Research NIHR Biomedical Research Centre.

Disclaimer
The views and opinions expressed therein are those of the authors and do not necessarily reflect those of the HTA programme, NIHR, NHS or the Department of Health and Social Care.

The funder and sponsor approve any amendments to the study but have no direct involvement in study design; collection, management, analysis, and interpretation of data; writing of the report; and the decision to Submit this report for publication.

Competing interests' statement
EL: reports personal fees from Abbott Molecular, personal fees from Glaxo Smith Kline, personal fees from Pfizer, personal fees from Norvatis, personal fees from Covidien, personal fees from Roche, personal fees from Lily Oncology, personal fees from Boehringer Ingelheim, personal fees from Medela, grants and personal fees from ScreenCell, personal fees from Ethicon, grants from Clearbridge Biomedics, grants from Illumina, grants from Guardant Health, personal fees from AstraZenecia (outside the submitted work), a patent P52435GB issued to Imperial Innovations, and a patent P57988GB issued to Imperial Innovations and Director of lung screening at Cromwell Hospital, CI for VIOLET NIHR HTA (13/04/03), CI for MARS 2 NIHR HTA (15/188/31); DAF: reports grants from Astex Therapeutics, personal fees from Aldeyra, grants from Boehringer Ingelheim, non-financial support from Clovis, non-financial support from Eli Lilly, from BMS, personal fees from Inventiva, personal fees from Paredox, personal fees and non-financial support from Roche, grants from MSD, grants from Bayer, during the conduct of the study; SP: reports personal fees from BMS, personal fees from Roche, personal fees from Takeda, personal fees from AstraZeneca, personal fees from Pfizer, personal fees from MSD, personal fees from EMD Serono, personal fees from Guardant Health, personal fees from Abbvie, personal fees from Boehringer Ingelheim, personal fees from OncLive, personal fees from Medscape, personal fees from Incyte (outside the submitted work); PK: reports non-Financial Support: Travel grant to ESMO Congress 2018, was sponsored by Boeringer Ingelheim, West Midlands UK Oncology Specialist Trainee Regional Training Day April 2018, meeting was sponsored by Roche, Servier and Bristol Myers Squibb in the purchase of exhibition stand space, Eisai was sponsoring this meeting towards the cost of catering. TM: reports other from MSD, other from Roche, other from Tesaro, from AstraZeneca, personal fees from Tesaro, personal fees

For peer review only - http://bmjopen.bmj.com/site/about/guidelines.xhtml

from Roche, personal fees from MSD, personal fees from Amgen, other from BMS (outside the submitted work); KA: reports grants from NIHR HTA, during the conduct of the study; RAH: reports grants from NIHR HTA, during the conduct of the study; KJ: reports grants from NIHR HTA, during the conduct of the study; ES: reports grants from UK National Institute for Health Research (Health Technology Assessment Programme), Department of Health and Social Care, during the conduct of the study; CAR: reports grants from National Institute for Health Research, during the conduct of the study.

Unless otherwise stated above, authors have declared no competing interests.

Patient Consent for publication
Not required.

Ethics Approval
This study received a favourable opinion by the London - Camberwell St. Giles Research Ethics Committee (REC; reference 13/LO/1481) on 7th November 2013.

Provenance and peer review
Not commissioned, externally peer reviewed.

Data sharing
Data will not be made available for sharing until after publication of the main results of the study. Thereafter, anonymised individual patient data will be made available for secondary research, conditional on prior consent having been given by the patients and assurance from the secondary researcher that the proposed use of the data is compliant with the Medical Research Council (MRC) Policy on Data Sharing regarding scientific quality, ethical requirements and value for money. A minimum requirement with respect to scientific quality will be a publicly available pre-specified protocol describing the purpose, methods and analysis of the secondary research, e.g. a protocol for a Cochrane systematic review. The second file containing patient identifiers would be retained for record linkage or a similar purpose, Subject to confirmation that the secondary research protocol has been approved by a UK REC or other similar, approved ethics review body.

ORCID IDs
Daisy Elliott: 0000-0001-8143-9549; Robert Rintoul: 0000-0003-3875-3780; Katherine Joyce: 0000-0002-5539-7178; Chris A Rogers: 0000-0002-9624-2615

References

1. Executive HaS. Asbestos-related disease statistics in Great Britain, 2019 2019.
2. Peto J, Hodgson JT, Matthews FE, et al. Continuing increase in mesothelioma mortality in Britain. *Lancet* 1995;345(8949):535-9.
3. National Institute of Health and Care Excellence. Pemetrexed for the treatment of malignant pleural mesothelioma. Technology appraisal guidance [TA135]. 2008
4. Vogelzang NJ, Rusthoven JJ, Symanowski J, et al. Phase III study of pemetrexed in combination with cisplatin versus cisplatin alone in patients with malignant pleural mesothelioma. *Journal of clinical oncology* 2003;21(14):2636-44.

For peer review only - http://bmjopen.bmj.com/site/about/guidelines.xhtml

5. Royal College of Physicians. National Lung Cancer Audit pleural mesothelioma report 2016 (for the audit period 2014). London: Royal College of Physicians, 2016.
6. Rice D, Rusch V, Pass H, et al. Recommendations for uniform definitions of surgical techniques for malignant pleural mesothelioma: a consensus report of the international association for the study of lung cancer international staging committee and the international mesothelioma interest group. *Journal of thoracic oncology* 2011;6(8):1304-12.
7. Treasure T, Lang-Lazdunski L, Waller D, et al. Extra-pleural pneumonectomy versus no extra-pleural pneumonectomy for patients with malignant pleural mesothelioma: clinical outcomes of the Mesothelioma and Radical Surgery (MARS) randomised feasibility study. *The Lancet Oncology* 2011;12(8):763-72. doi: 10.1016/S1470-2045(11)70149-8
8. Rintoul RC, Ritchie AJ, Edwards JG, et al. Efficacy and cost of video-assisted thoracoscopic partial pleurectomy versus talc pleurodesis in patients with malignant pleural mesothelioma (MesoVATS): an open-label, randomised, controlled trial. *Lancet* 2014;384(9948):1118-27. doi: 10.1016/S0140-6736(14)60418-9
9. Cao C, Tian DH, Pataky KA, et al. Systematic review of pleurectomy in the treatment of malignant pleural mesothelioma. *Lung Cancer* 2013;81(3):319-27. doi: 10.1016/j.lungcan.2013.04.024
10. Lang-Lazdunski L, Bille A, Lal R, et al. Pleurectomy/decortication is superior to extrapleural pneumonectomy in the multimodality management of patients with malignant pleural mesothelioma. *Journal of Thoracic Oncology* 2012;7(4):737-43. doi: 10.1097/JTO.0b013e31824ab6c5
11. Teh E, Fiorentino F, Tan C, et al. A systematic review of lung-sparing extirpative surgery for pleural mesothelioma. *Journal of the Royal Society of Medicine* 2011;104(2):69-80. doi: 10.1258/jrsm.2010.100345
12. Donovan JL, Rooshenas L, Jepson M, et al. Optimising recruitment and informed consent in randomised controlled trials: the development and implementation of the Quintet Recruitment Intervention (QRI). *Trials* 2016;17(1):283. doi: 10.1186/s13063-016-1391-4
13. Rooshenas L, Paramasivan S, Jepson M, et al. Intensive Triangulation of Qualitative Research and Quantitative Data to Improve Recruitment to Randomized Trials: The QuinteT Approach. *Qual Health Res* 2019;29(5):672-79. doi: 10.1177/1049732319828693 [published Online First: 2019/02/23]
14. Rooshenas L, Scott LJ, Blazeby JM, et al. The QuinteT Recruitment Intervention supported five randomized trials to recruit to target: a mixed-methods evaluation. *J Clin Epidemiol* 2019;106:108-20. doi: 10.1016/j.jclinepi.2018.10.004 [published Online First: 2018/10/20]
15. EuroQol Group. EuroQol - a new facility for the measurement of health-related quality of life. *Health policy* 1990;16(3):199-208.
16. Herdman M, Gudex C, Lloyd A, et al. Development and preliminary testing of the new five-level version of EQ-5D (EQ-5D-5L). *Quality of Life Research* 2011;20(10):1727-36. doi: 10.1007/s11136-011-9903-x [published Online First: 2011/04/12]
17. Utley M, Fiorentino F, Treasure T. Obtaining an upper estimate of the survival benefit associated with surgery for mesothelioma. *European Journal of Cardiothoracic Surgery* 2010;38:241-4.
18. Briel M, Olu KK, von Elm E, et al. A systematic review of discontinued trials suggested that most reasons for recruitment failure were preventable. *Journal of Clinical Epidemiology* 2016;80:8-15. doi: 10.1016/j.jclinepi.2016.07.016 [published Online First: 2016/08/09]
19. Blencowe NS, Cook JA, Pinkney T, et al. Delivering successful randomized controlled trials in surgery: Methods to optimize collaboration and study design. *Clinical Trials* 2017;14(2):211-18. doi: 10.1177/1740774516687272 [published Online First: 2017/02/01]
20. National Institute for Health and Care Excellence. Guide to the Methods of Technology Appraisal 2013. London: National Institute for Health and Care Excellence, 2013.
21. National Institute for Health and Care Excellence. National Institute for Health and Care Excellence. Position statement on use of the EQ-5D-5L valuation set for England (updated October 2019) [updated October 2019. Available from:

For peer review only - http://bmjopen.bmj.com/site/about/guidelines.xhtml

https://www.nice.org.uk/about/what-we-do/our-programmes/nice-guidance/technology-appraisal-guidance/eq-5d-5l accessed 18 December 2019.

22. White IR, Royston P, Wood AM. Multiple imputation using chained equations: Issues and guidance for practice. *Stat Med* 2011;30(4):377-99.

For peer review only

For peer review only - http://bmjopen.bmj.com/site/about/guidelines.xhtml

Figure 1 **Trial schema showing the recruitment pathway for the MARS 2 study**

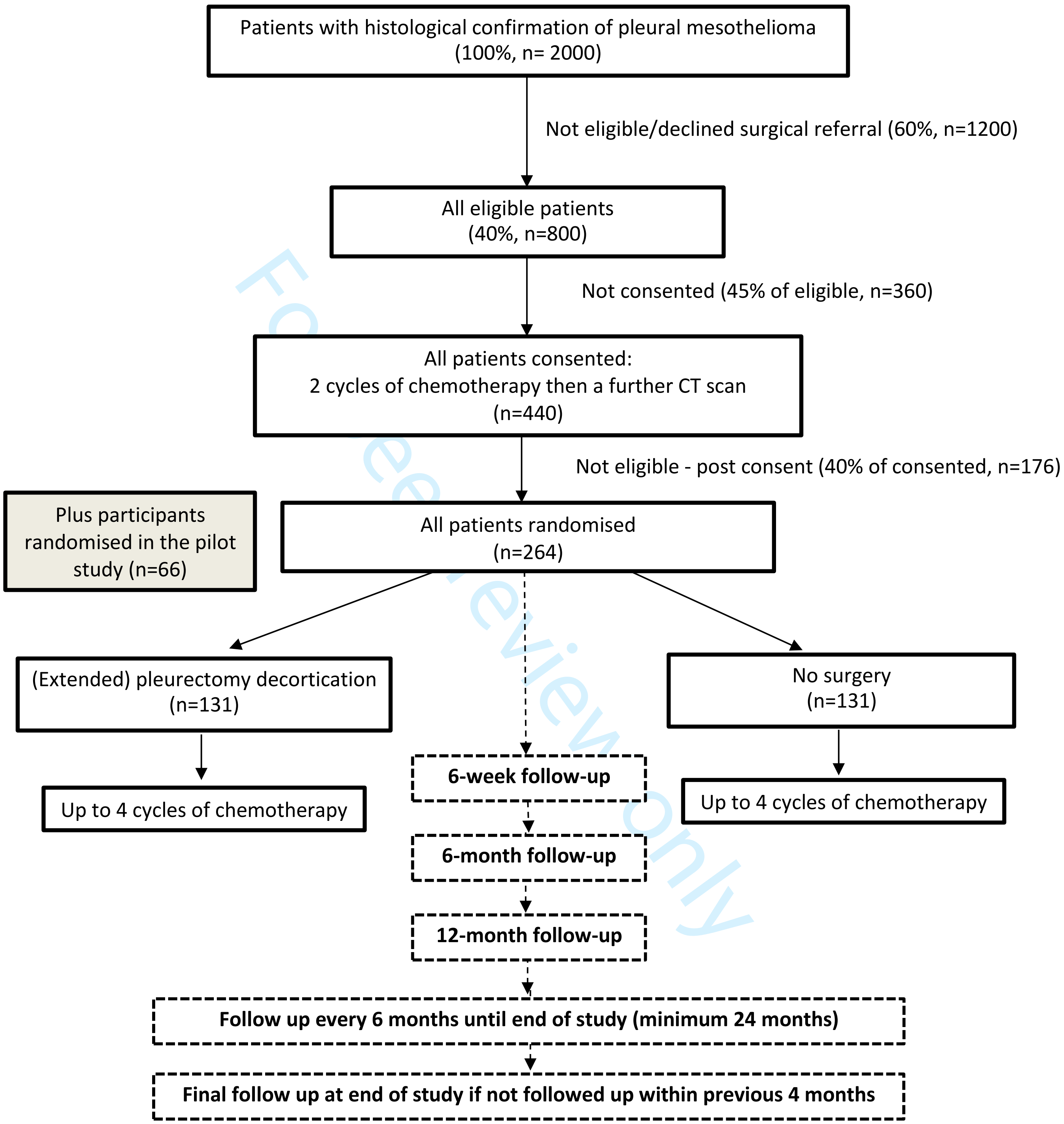

For peer review only - http://bmjopen.bmj.com/site/about/guidelines.xhtml

STANDARD PROTOCOL ITEMS: RECOMMENDATIONS FOR INTERVENTIONAL TRIALS

SPIRIT 2013 Checklist: Recommended items to address in a clinical trial protocol and related documents*

Section/item	Item No	Description	Addressed on page number
**Administrative information**			
Title	1	Descriptive title identifying the study design, population, interventions, and, if applicable, trial acronym	1
Trial registration	2a	Trial identifier and registry name. If not yet registered, name of intended registry	4
	2b	All items from the World Health Organization Trial Registration Data Set	N/A
Protocol version	3	Date and version identifier	16
Funding	4	Sources and types of financial, material, and other support	23
Roles and responsibilities	5a	Names, affiliations, and roles of protocol contributors	1-3,23
	5b	Name and contact information for the trial sponsor	18
	5c	Role of study sponsor and funders, if any, in study design; collection, management, analysis, and interpretation of data; writing of the report; and the decision to submit the report for publication, including whether they will have ultimate authority over any of these activities	24
	5d	Composition, roles, and responsibilities of the coordinating centre, steering committee, endpoint adjudication committee, data management team, and other individuals or groups overseeing the trial, if applicable (see Item 21a for data monitoring committee)	N/A

For peer review only

**Introduction**			
Background and rationale	6a	Description of research question and justification for undertaking the trial, including summary of relevant studies (published and unpublished) examining benefits and harms for each intervention	5,6
	6b	Explanation for choice of comparators	6
Objectives	7	Specific objectives or hypotheses	6,7
Trial design	8	Description of trial design including type of trial (eg, parallel group, crossover, factorial, single group), allocation ratio, and framework (eg, superiority, equivalence, noninferiority, exploratory)	7
**Methods: Participants, interventions, and outcomes**			
Study setting	9	Description of study settings (eg, community clinic, academic hospital) and list of countries where data will be collected. Reference to where list of study sites can be obtained	7,8
Eligibility criteria	10	Inclusion and exclusion criteria for participants. If applicable, eligibility criteria for study centres and individuals who will perform the interventions (eg, surgeons, psychotherapists)	8
Interventions	11a	Interventions for each group with sufficient detail to allow replication, including how and when they will be administered	8,9
	11b	Criteria for discontinuing or modifying allocated interventions for a given trial participant (eg, drug dose change in response to harms, participant request, or improving/worsening disease)	8,9
	11c	Strategies to improve adherence to intervention protocols, and any procedures for monitoring adherence (eg, drug tablet return, laboratory tests)	7,8, 13,14
	11d	Relevant concomitant care and interventions that are permitted or prohibited during the trial	9
Outcomes	12	Primary, secondary, and other outcomes, including the specific measurement variable (eg, systolic blood pressure), analysis metric (eg, change from baseline, final value, time to event), method of aggregation (eg, median, proportion), and time point for each outcome. Explanation of the clinical relevance of chosen efficacy and harm outcomes is strongly recommended	9-11
Participant timeline	13	Time schedule of enrolment, interventions (including any run-ins and washouts), assessments, and visits for participants. A schematic diagram is highly recommended (see Figure)	11

For peer review only - http://bmjopen.bmj.com/site/about/guidelines.xhtml

Sample size	14	Estimated number of participants needed to achieve study objectives and how it was determined, including clinical and statistical assumptions supporting any sample size calculations	12,13
Recruitment	15	Strategies for achieving adequate participant enrolment to reach target sample size	12-14
**Methods: Assignment of interventions (for controlled trials)**			
Allocation:			
Sequence generation	16a	Method of generating the allocation sequence (eg, computer-generated random numbers), and list of any factors for stratification. To reduce predictability of a random sequence, details of any planned restriction (eg, blocking) should be provided in a separate document that is unavailable to those who enrol participants or assign interventions	9
Allocation concealment mechanism	16b	Mechanism of implementing the allocation sequence (eg, central telephone; sequentially numbered, opaque, sealed envelopes), describing any steps to conceal the sequence until interventions are assigned	9
Implementation	16c	Who will generate the allocation sequence, who will enrol participants, and who will assign participants to interventions	9
Blinding (masking)	17a	Who will be blinded after assignment to interventions (eg, trial participants, care providers, outcome assessors, data analysts), and how	11
	17b	If blinded, circumstances under which unblinding is permissible, and procedure for revealing a participant's allocated intervention during the trial	N/A
**Methods: Data collection, management, and analysis**			
Data collection methods	18a	Plans for assessment and collection of outcome, baseline, and other trial data, including any related processes to promote data quality (eg, duplicate measurements, training of assessors) and a description of study instruments (eg, questionnaires, laboratory tests) along with their reliability and validity, if known. Reference to where data collection forms can be found, if not in the protocol	10,11,12
	18b	Plans to promote participant retention and complete follow-up, including list of any outcome data to be collected for participants who discontinue or deviate from intervention protocols	14-16

Data management	19	Plans for data entry, coding, security, and storage, including any related processes to promote data quality (eg, double data entry; range checks for data values). Reference to where details of data management procedures can be found, if not in the protocol	N/A
Statistical methods	20a	Statistical methods for analysing primary and secondary outcomes. Reference to where other details of the statistical analysis plan can be found, if not in the protocol	14,15
	20b	Methods for any additional analyses (eg, subgroup and adjusted analyses)	15
	20c	Definition of analysis population relating to protocol non-adherence (eg, as randomised analysis), and any statistical methods to handle missing data (eg, multiple imputation)	14
**Methods: Monitoring**			
Data monitoring	21a	Composition of data monitoring committee (DMC); summary of its role and reporting structure; statement of whether it is independent from the sponsor and competing interests; and reference to where further details about its charter can be found, if not in the protocol. Alternatively, an explanation of why a DMC is not needed	22
	21b	Description of any interim analyses and stopping guidelines, including who will have access to these interim results and make the final decision to terminate the trial	N/A
Harms	22	Plans for collecting, assessing, reporting, and managing solicited and spontaneously reported adverse events and other unintended effects of trial interventions or trial conduct	N/A
Auditing	23	Frequency and procedures for auditing trial conduct, if any, and whether the process will be independent from investigators and the sponsor	N/A
**Ethics and dissemination**			
Research ethics approval	24	Plans for seeking research ethics committee/institutional review board (REC/IRB) approval	15,16
Protocol amendments	25	Plans for communicating important protocol modifications (eg, changes to eligibility criteria, outcomes, analyses) to relevant parties (eg, investigators, REC/IRBs, trial participants, trial registries, journals, regulators)	16

Consent or assent	26a	Who will obtain informed consent or assent from potential trial participants or authorised surrogates, and how (see Item 32)	9
	26b	Additional consent provisions for collection and use of participant data and biological specimens in ancillary studies, if applicable	N/A
Confidentiality	27	How personal information about potential and enrolled participants will be collected, shared, and maintained in order to protect confidentiality before, during, and after the trial	25
Declaration of interests	28	Financial and other competing interests for principal investigators for the overall trial and each study site	23, 24
Access to data	29	Statement of who will have access to the final trial dataset, and disclosure of contractual agreements that limit such access for investigators	25
Ancillary and post-trial care	30	Provisions, if any, for ancillary and post-trial care, and for compensation to those who suffer harm from trial participation	N/A
Dissemination policy	31a	Plans for investigators and sponsor to communicate trial results to participants, healthcare professionals, the public, and other relevant groups (eg, via publication, reporting in results databases, or other data sharing arrangements), including any publication restrictions	4, 16
	31b	Authorship eligibility guidelines and any intended use of professional writers	N/A
	31c	Plans, if any, for granting public access to the full protocol, participant-level dataset, and statistical code	N/A
**Appendices**			
Informed consent materials	32	Model consent form and other related documentation given to participants and authorised surrogates	On request
Biological specimens	33	Plans for collection, laboratory evaluation, and storage of biological specimens for genetic or molecular analysis in the current trial and for future use in ancillary studies, if applicable	N/A

*It is strongly recommended that this checklist be read in conjunction with the SPIRIT 2013 Explanation & Elaboration for important clarification on the items. Amendments to the protocol should be tracked and dated. The SPIRIT checklist is copyrighted by the SPIRIT Group under the Creative Commons "Attribution-NonCommercial-NoDerivs 3.0 Unported" license.

For peer review only - http://bmjopen.bmj.com/site/about/guidelines.xhtml
